# Enantioselective Total Synthesis of (*R,R*)-Blumenol B and d_9_-(*R,R*)-Blumenol B

**DOI:** 10.3390/molecules27217294

**Published:** 2022-10-27

**Authors:** Shi Min Tan, Shaun W. P. Rees, Rebecca E. Jelley, Jin Wang, Bruno Fedrizzi, David Barker

**Affiliations:** 1 School of Chemical Sciences, University of Auckland, 23 Symonds St., Auckland 1010, New Zealand; 2 Centre for Green Chemical Science, School of Chemical Sciences, University of Auckland, Private Bag 92019, Auckland 1010, New Zealand; 3 MacDiarmid Institute for Advanced Materials and Nanotechnology, Victoria University of Wellington, Wellington 6012, New Zealand

**Keywords:** wine aroma, C_13_-norisoprenoids, blumenol B, isotopic labelling

## Abstract

C_13_-norisoprenoids are of particular importance to grapes and wines, as these molecules influence wine aroma and have been shown to significantly contribute to the distinct character of various wine varieties. Blumenol B is a putative precursor to a number of important wine aroma compounds, including the well-known compounds theaspirone and vitispirane. The enantioselective synthesis of (*R*,*R*)-blumenol B from commercially available 4-oxoisophorone was achieved using a short and easily scaleable route, which was then successfully applied to the synthesis of poly-deuterated d9-blumenol B.

## 1. Introduction

Norisoprenoids are molecules found in grapes and wines that result from the direct degradation of carotenoids either photochemically, chemically or via oxidase-coupled mechanisms [1,2]. Those with 13 carbon atoms, i.e., C_13_-norisoprenoids, are of particular importance as these molecules, even at very low concentrations, are key contributors to the aroma profile of wine. C_13_-norisoprenoids are known to influence the distinctive sensory character of many wine varieties including Cabernet Sauvignon, Chenin blanc, Sauvignon blanc, Syrah and Riesling [3,4,5].

C_13_-norisoprenoids such as β-damascenone, α-ionol, β-ionol, α-ionone, β-ionone, vitispirane, actinidol, vomifoliol, and TDN (1,1,6-trimethyl-1,2-dihydronaphthalene) have been studied extensively not only in grapes and wines [6,7,8,9], but in other natural sources including honey, fruit and essential oils [1,10,11]; on the other hand, studies of blumenol B remain scarce.

Blumenol B **1** (also known as 7,8-dihydrovomifoliol), in its aglycone form, has been identified following the extraction and subsequent enzymatic hydrolysis of the non-volatile glycosylate precursor, icariside B5, which is present in the grapes and wines of Weisser Riesling [4], and also in Riesling grapevine leaves [3]. Blumenol B **1** has also been obtained via glycosylated precursor iscariside B5 from other natural sources, including *Pinus sylvestris* and *Picea abies* needles [12], *Casearia sylvestris* leaves [13], and *Sarcandra glabra Nakai* [14]. Furthermore, blumenol B **1** has been directly isolated from the leaves of *Podocarpus blumei* [15], and *Cannabis sativa* [16].

**Scheme 1:**
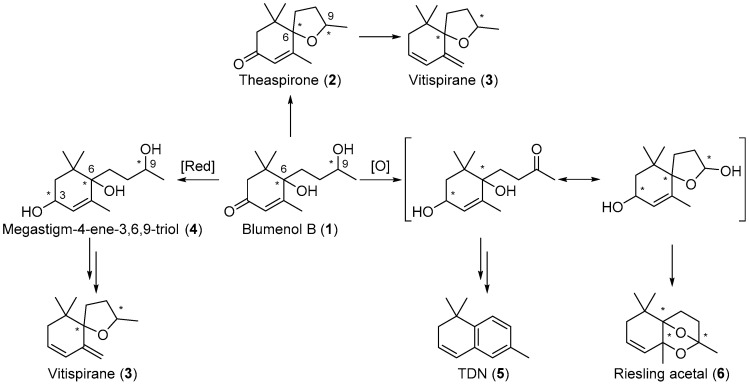
Blumenol B (**1**) is a precursor in the formation of theaspirone (**2**) and other important C_13_-norisoprenoids including vitispirane (**3**), megastigm-4-ene-3,6,9-triol (**4**), TDN (**5**) and Riesling acetal (**6**) in grapes and wines.

Blumenol B **1** is reported to be a precursor for other important aroma compounds and, in its reduced and oxidised forms, has been reported to prompt the formation of other C_13_-norisoprenoids, including theaspirone **2** [17], vitispirane **3** [18], megastigm-4-ene-3,6,9-triol **4**, 1,1,6-trimethyl-1,2-dihydronaphthalene (TDN) **5** [19], and Riesling acetal **6** (Scheme 1) [20]. Stereochemistry is important in these aroma compounds as the various stereoisomers of these compounds contribute to the distinct aroma profiles of various grapes and wines [6,7,21,22,23,24]. For example, the (*S*,*S*)-stereoisomer of theaspirone **2** imparts an earthy scent, while the enantiomer gives off a sweet and tea-like odour [18,25,26]. The racemic mixture of vitispirane **3** is reported to have a woody, balsamic, resinous, and spicy aroma profile [18,27]. Pure isomers (*R*,*R*)- and (*S*,*S*)-vitispirane exert a floral and fruity aroma, while (*S*,*R*)- and (*R*,*S*)-vitispirane imparts a strong scent of exotic flowers and woody tone [6]. The aroma profile of another aroma compound, Riesling acetal **6**, also differs between its enantiomeric forms, (+)-Riesling acetal imparts a subtle floral, fruity and woody scent, while (−)-Riesling acetal gives off a weak floral and camphoraceous aroma [1,19,28]. It is proposed that the stereochemistry of the precursor blumenol B **1** is important in the formation of the various isomers of **2**, **3**, and **6**, and is thus important in the overall aroma profile of the wines.

To date, blumenol B **1** has only been synthesized via routes that were either non-stereospecific, lengthy (17-steps) or involved a semi-synthetic route from complex isolated natural products, such as the case of (*S,R*)- and (*R,S*)-blumenol B [17,25,29]. We wished to develop an efficient stereospecific synthesis of blumenol B from commercially available materials that would also be suitable for the preparation of isotopically labelled standards. Such standards could be employed for the identification and quantification of aglycones that are enzymatically and/or biochemically released from glycosidic precursors found in wine.

## 2. Results

**Scheme 2:**
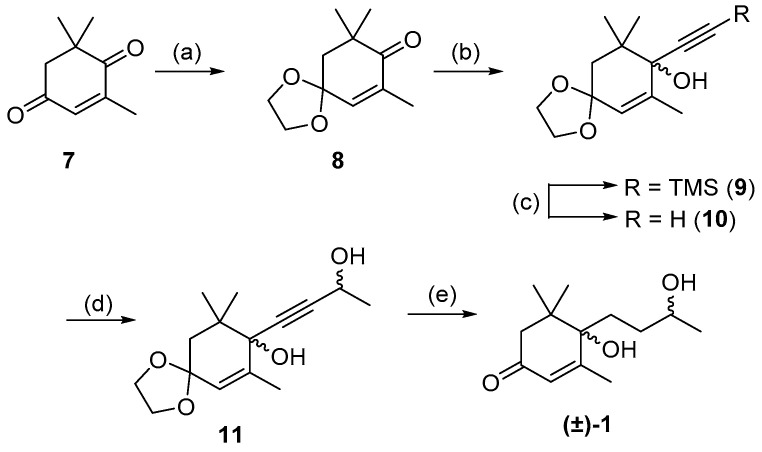
Synthesis of isomeric mixture of (±)-blumenol B **1**. Reagents and conditions: (**a**) Ethylene glycol, *p*-TsOH, toluene, 24 h, 86%; (**b**) Trimethylsilylacetylene, *^n^*BuLi, THF, −78 °C, 16 h, 75%; (**c**) K_2_CO_3_, MeOH, 1 h, 64%; (**d**) Acetaldehyde, LDA, THF, −78 °C, 14 h, 62%; (**e**) Lindlar catalyst, H_2_, MeOH, 20 h then 2 M HCl, THF, 18 h, 52%.

A previous synthesis of structurally similar C_13_-norisoprenoids such as (±)-theaspirone and (±)-(Z)-vomifoliol utilized the coupling of a organometallic reagent with a cyclic ketone to form the key carbon–carbon bond at the tertiary alcohol center [17,25]. These previously endeavors were only able to form ring-opened derivatives such as **1** and **4** by an acidic ring cleavage of spiro-like molecules similar to **2**. This not only resulted in ring-opened compounds but numerous rearranged and dehydrated species. We aimed to employ a similar organometallic strategy of addition to a ketone when attempting the racemic synthesis of blumenol B **1,** but would avoid the formation of spiro-compounds enroute to **1**. We began using the commercially available 4-oxoisophorone **7** as the substrate for coupling with a lithiated acetylide. The first step in this pathway was protection of the less hindered carbonyl in 4-oxoisophorone **7** to afford the known ketal **8**, which was reacted with lithiated TMS-acetylene giving tertiary alcohol **9** in 65% yield across the two steps (Scheme 2) [30,31]. Removal of the TMS-group formed the free acetylide **10**, which was reacted with acetaldehyde to afford diol **11** as an inseparable mixture of diastereomers. Acetylene **11** then underwent exhaustive hydrogenation of the exocyclic alkyne, followed by deprotection of the ketone acetal to give (±)-blumenol B **1** as an inseparable 2:1 diastereomeric mixture in 53% yield over two steps. It was found that hydrogenation for 20 h reaction time resulted in an exclusive reactive of the endocyclic alkene [32,33]. However, longer reaction times were found to result in unwanted over-reduction. The spectroscopic data of the mixture of isomers of (±)-**1** were in agreement with those reported by Matsunami et al. [29] (Tables S1 and S2).

**Scheme 3:**
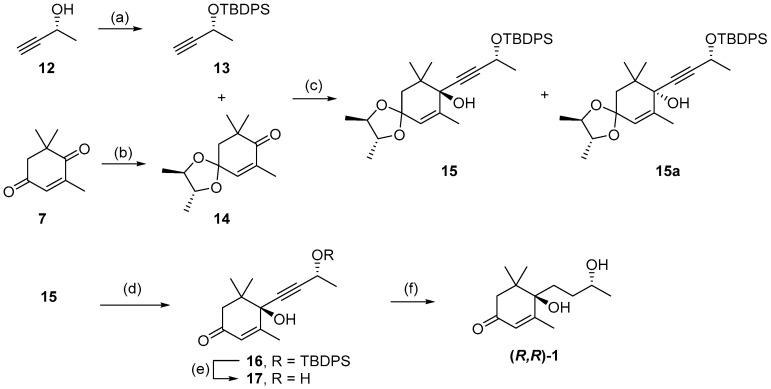
Synthesis of (*R*,*R*) blumenol B **1**. Reagents and conditions; (**a**) TBDPS-Cl, imidazole, DMF, 0 °C, overnight, quant.; (**b**) 2*R*,3*R*-(-)-2,3-butanediol, *p*-TsOH, toluene, overnight, 93%; (**c**) *^n^*BuLi, THF, −78 °C, 14 h, 47% **15** and 12% **15a**; (**d**) 2 M HCl, THF, 18 h, 95%; (**e**) TBAF, THF, 0 °C, 15 h, 96%; (**f**) Pd/BaSO_4_, H_2_, MeOH, 19 h, 57%.

Following the development of this short racemic synthesis of **1**, the next goal was the stereoselective synthesis of (*R,R*)-blumenol B using enantiopure reagents to direct the stereochemistry of the two chiral centers (Scheme 3). The first enantiopure reagent employed was commercially available (*R*)-(+)-3-butyn-2-ol **12**, which was protected with a TBDPS group giving **13** in quantitative yield [34]. The second enantiopure reagent used was 2*R*,3*R*-(-)-2,3-butanediol, which was used to protect the less-hindered carbonyl on 4-oxoisophorone **7**, forming the known chiral ketal **14** [35]. Alkynylation of ketal **14** using lithiated **13** afforded a 1.2:1 diastereomeric mixture of the *R*- and *S*- configuration at the newly formed tertiary alcohol stereocenter. Subsequent recrystallization using petroleum ether gave the major *R*-isomer **15** in 47% yield as a white solid and minor *S*-isomer **15a** in 12% yield as a colourless oil, allowing for **15** to be easily separated. Similar facial selectivity, and solid versus oil for the two diastereoisomers, has been observed for the additional of alternative acetylenes with chiral ketone **14**, which allowed for the determination of absolute stereochemistry in **15** [35]. This was further confirmed when crystals of d_9_-**16** were formed (see below) and X-ray unequivocally determined the absolute stereochemistry. With **15** formed, both the acetal group and TBDPS-protecting group were removed to form propargyl alcohol **17** in 91% yield, across the two steps. The hydrogenation of propargyl alcohol **17** in the presence of Lindlar catalyst afforded (*R*,*R*)-blumenol B **1** in 57% yield, which exhibited identical ^1^H and ^13^C NMR data to reported values for (*S*,*S*)-blumenol B (Table S3) [29].

**Scheme 4:**
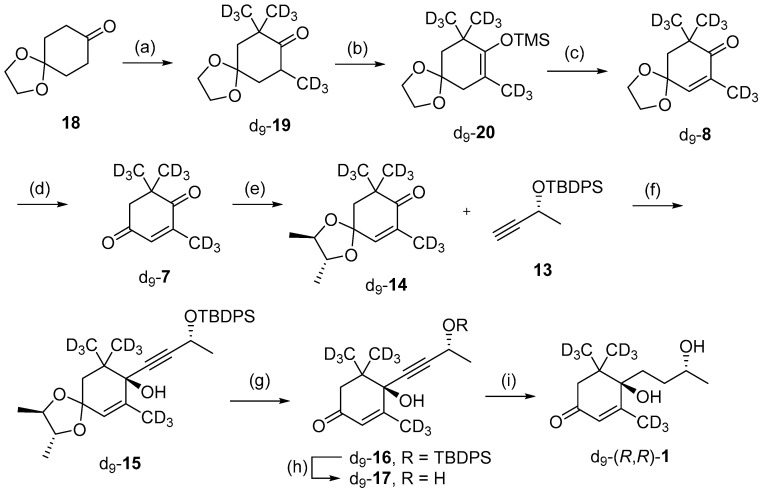
Synthesis of d_9_-(*R*,*R*)-blumenol B **1**. Reagents and conditions; (**a**) NaH, CD_3_I, THF, 0 °C—reflux, overnight, 77%; (**b**) LDA, Et_3_N, TMS-Cl, THF, −78 °C—r.t., overnight, 82%; (**c**) 10% Br_2_ in CH_2_Cl_2_, DBU, CH_2_Cl_2_. 0 °C, overnight, 85%; (**d**) 2M HCl, THF, r.t., overnight, quant.; (**e**) 2*R*,3*R*-(-)-2,3-butanediol, *p*-TsOH, toluene, overnight, 89%; (**f**) *^n^*BuLi, THF, −78 °C, overnight, 51%; (**g**) 2 M HCl, THF, r.t., 18 h, 94%; (**h**) TBAF, THF, 0 °C, 15 h, quant.; (**i**) Pd/BaSO_4_, H_2_, MeOH, 8 h, 44%.

Following the successful synthesis of (*R,R*)-blumenol B **1**, the next goal was to apply this methodology to synthesize isotopically labelled (*R,R*)-**1**, for possible use as an analytical standard for quantifying (*R,R*)-**1**. This would require the synthesis of an isotopically labelled precursor and it was decided to use the previously reported d_9_-**8** in this study [36]. The synthesis began with treating 1,4-cyclohexanedione monoethylene acetal **18** with four equivalents of CD_3_I, which afforded the target tri-substituted d_9_-**19** in 77% (Scheme 4). Next, the α,β-unsaturated ketone was installed in two steps by converting d_9_-**19** to the TMS-protected enol d_9_-**20**, which was then subjected to one-pot halogenation and elimination, using Br_2_ followed by DBU to give the target enone d_9_-**8** in 70% yield over the two steps. Subsequent removal of the acetal group using 2M HCl afforded d_9_-**7**, which could then be utilized for synthesis of d_9_-(*R*,*R*) blumenol B **1** via the aforementioned methods. In brief, d_9_-7 was protected using 2*R*,3*R*-(-)-2,3-butanediol to give chiral ketal d_9_-**14**, which was then reacted with acetylide **13** affording d_9-_**15** in 44% yield over the two steps. Chiral acetal d_9_-**15** was removed using 2M HCl and subsequent TBDPS deprotection with TBAF gave d_9_-**17** in 94% yield. X-ray diffraction studies on TBDPS-protected d_9-_**16** intermediate confirmed the desired *R,R* diastereomer had formed (Figure S1). Lastly, the alkyne motif was fully hydrogenated using H_2_ and Lindlar catalyst, forming d_9_-(*R,R*) blumenol B **1** in 44% yield.

## 3. Materials and Methods

### 3.1. General Experimental Details

All reactions in non-aqueous solvents were carried out under an inert atmosphere using anhydrous AR grade solvents. Solvents used for reaction work up and purification were used as purchased, without further purification. Thin-layer chromatography (TLC) was performed using Merck silica gel F354 aluminium plates pre-coated with silica. Flash chromatography was carried out using Silica Gel 60 (40–63 μm, 230–430 mesh ASTM) utilising solvent systems defined in the experimental procedure for each synthesized molecule. Infra-red (IR) spectra were obtained using a Perkin-Elmer Spectrum 1000 series Fourier Transform Infra-Red ATR spectrometer. Melting points were measured using a Reicher-Kofler block and are uncorrected. NMR spectra were obtained using a Bruker Avance DRX 400 MHz spectrometer at ambient temperature. Chemical shifts are reported relative to the residual solvent peak of either CDCl_3_ (δ 7.26 for ^1^H and δ 77.16 for ^13^C) or CD_3_OD (δ 3.31 for ^1^H and δ 49.00 for ^13^C). ^1^H NMR data are reported in the following sequence: position (δ), relative integral, multiplicity (s, singlet; d, doublet; t, triplet; q, quartet; dd, doublet of doublets; m, multiplet; br s, broad singlet; br d, broad doublet), coupling constant (*J*, Hz), and proton assignment. ^13^C NMR data were reported in the following sequence: position (δ), multiplicity (d, doublet; q, quartet), coupling constant (*J*, Hz), and the carbon assignment. NMR assignments were made using a combination of ^1^H NMR, ^13^C NMR, HSQC and HMBC experiments. High-resolution mass spectroscopy (HRMS) was carried out using electrospray ionisation (ESI) on a MicroTOF-Q mass spectrometer.

### 3.2. Synthesis of Compounds

4,4-(Ethylenedioxy)-2,6,6-trimethylcyclohex-2-en-1-one **8**: A mixture of 4-oxoisophorone **7** (3.0 g, 19.7 mmol), ethylene glycol (1.46 mL, 26.2 mmol), and toluene-*p*-sulfonic acid monohydrate (0.09 g, 0.473 mmol) in toluene (14.8 mL) were heated at reflux overnight with a Dean-Stark trap. The solution was left to cooled and quenched with sat. aq. NaHCO_3_ (15 mL) and was extracted with diethyl ether (3 × 15 mL). The combined organic extracts were washed with water and dried over anhydrous MgSO_4_. The solvent was removed in vacuo and the crude product was purified by flash chromatography (1:1 hexanes, ethyl acetate) to yield the *title compound* **8** (3.325 g, 86%) as a yellow oil. **R_F_** (3:1 petroleum ether, ethyl acetate) = 0.54. **δ_H_** (400 MHz; CDCl_3_) 1.20 (6H, s, 6-(CH_3_)_2_), 1.79 (3H, d, *J* = 1.39 Hz, 2-CH_3_), 2.08 (2H, s, 5-H), 3.98–4.02 (4H, m, OCH_2_CH_2_O), 6.30 (1H, br s, 3-H). The spectroscopic data were in agreement with the literature values [31].

4,4-(Ethylenedioxy)-2,6,6-trimethyl-1-trimethylsilanyl-ethynylcyclohex-2-en-1-ol **(±)-9:** To a stirred solution of trimethylsilylacetylene (0.29 mL, 2.03 mmol) in THF (3 mL) under an nitrogen atmosphere at −78 °C was added *^n^*BuLi (1.6 M in hexanes, 0.22 mL, 2.03 mmol). The reaction mixture was stirred at −78 °C for 15 min and a solution of **8** (0.20 mg, 1.01 mmol) in THF (6 mL) was added dropwise to the previously prepared reaction mixture. The resultant mixture was then stirred under a nitrogen atmosphere for 1 h, and the reaction was quenched with sat. aq. NH_4_Cl (5 mL), and was extracted with diethyl ether (3 × 5 mL), before the combined organic extracts were washed with water (5 mL), brine (5 mL) and dried over anhydrous MgSO_4_ and the solvent was removed in vacuo. The crude product was purified by flash chromatography (14:1 hexanes, ethyl acetate) to yield the *title compound* **(±)-9** (0.22 g, 75%) as a yellow oil. **R_F_** (14:1, 9:1 hexanes, ethyl acetate) = 0.39. **δ_H_** (400 MHz; CDCl_3_) 0.16 (9H, s, Si(CH_3_)_3_), 1.05 and 1.12 (each 3H, s, 6-(CH_3_)_2_), 1.89 (2H, s, 5-H), 1.91 (3H, s, 2-CH_3_), 3.90–3.95 (4H, m, 4H, m, OCH_2_CH_2_O), 5.36 (1H, br s, 3-H). The spectroscopic data were in agreement with the literature values [31].

4,4-(Ethylenedioxy)-2,6,6-trimethyl-1-ethynylcyclohex-2-en-1-ol **(±)-10**: To a stirred solution of **(±)-9** (1.80 g, 6.12 mmol) in methanol (50 mL), potassium carbonate (2.54 g, 18.8 mmol) was added, and the reaction mixture was stirred at room temperature for 1 h. The precipitate was filtered, the solvent was removed in vacuo, and was extracted with diethyl ether (3 × 50 mL); then, the combined organic extracts were washed with water, dried over anhydrous MgSO_4_ and the solvent removed in vacuo. The crude product was recrystallised from hexanes to yield the *title compound* **(±)-10** (1.85 g, 64%) as white needles. R_F_ (9:1 hexanes, ethyl acetate) = 0.47. Melting point: 76–78 °C (lit. 78–80 °C). δ_H_ (400 MHz; CDCl_3_) 1.10 (3H, s, 6-CH_3_), 1.16 (3H, s, 6-CH_3_), 1.88 (1H, d, *J* = 14.3 Hz, 5-H_a_), 1.93 (3H, d, *J* = 1.3 Hz, 2-CH_3_), 1.96 (1H, d, *J* = 14.2 Hz, 5-H_b_), 2.02 (1H, s, OH), 2.50 (1H, s, ethynyl CH), 3.90–3.96 (4H, m, OCH_2_CH_2_O), 5.38 (1H, s, 3-H). The spectroscopic data and melting point were in agreement with the literature values [31].

8-(3-Hydroxybut-1-yn-1yl)-7,9,9-trimethyl-1,4-dioxaspiro [4.5]dec-6-en-8-ol **(±)-11**: To a stirred solution of **(±)-10** (0.85g, 3.8 mmol) in THF (25 mL) under anitrogen atmosphere, LDA (5.71 mL, 11.4 mmol) was added at −78 °C, and the resultant mixture was stirred for 30 min. Acetaldehyde (0.327 mL, 5.85 mmol) in THF (12 mL) was added dropwise to the reaction mixture at −78 °C and was left to stir for 30 min; it was then further stirred at 0 °C under a nitrogen atmosphere overnight. The reaction mixture was quenched with sat. aq. NH_4_Cl (15 mL) and extracted with ethyl acetate (3 × 25 mL). The combined organic extracts were washed with brine (25 mL), dried over anhydrous MgSO_4_ and the solvent was removed in vacuo. The crude product was purified by flash chromatography (1:1 hexanes, ethyl acetate) to yield the *title compound* **(±)-10** (0.624 g, 62%) as a white solid. R_F_ (1:1 hexanes, ethyl acetate) = 0.3. Melting point: 135–138 °C. δ_H_ (400 MHz; CDCl_3_) 1.09 (3H, s, 9-CH_3_), 1.14 (3H, s, 9-CH_3_), 1.45 (3H, d, *J* = 6.5 Hz, CH(OH)C*H*_3_), 1.91 (3H, s, 7-CH_3_), 2.03 (2H, br d, *J* = 7.6 Hz, 5CH_2_), 3.90–3.97 (4H, m, OCH_2_CH_2_O), 4.54–4.57 (1H, m, C*H*(OH)CH_3_), 5.36 (1H, br s, 3-H). The ^1^H NMR spectroscopic data and melting point were in agreement with the literature values [25].

(±)-Blumenol B **(±)-1**: To a stirred solution of **(±)-11** (300 mg, 1.13 mmol) in MeOH (30 mL), Pa/BaSO_4_ (120 mg, 40% w/w) was added, and was stirred under a hydrogen atmosphere at room temperature for 18–20 h. The mixture was filtered through Celite^®^, washed with MeOH and the solvent was removed in vacuo to yield the alkane (160 mg, 53%) as a colorless oil, which was immediately dissolved in THF (8 mL). The solution was placed under a nitrogen atmosphere, 2 M HCl (0.8 mL) was added, and the resultant mixture was stirred at room temperature overnight. The solvent was removed in vacuo and extracted with ethyl acetate (3 × 5 mL), and the combined organic extracts were washed with brine (5 mL), and dried over anhydrous MgSO_4_, and the solvent was *removed in vacuo*. The crude product was purified by flash chromatography (1:3 hexanes, ethyl acetate) to yield the *title compound* **(±)-1** (153 mg, 96%) as a colorless oil. R_F_ (1:1 hexanes, ethyl acetate) = 0.15. δ_H_ (400 MHz; CD_3_OD) 1.02 (3H, s, 1-H), 1.10 (3H, s, 12-H), 1.22 (3H, d, *J* = 6.0 Hz, 10-H), 1.40–1.48 (1H, m, 8-H), 1.68–1.72 (1H, m, 8-H), 1.75–1.83 (1H, m, 7-H), 1.92–1.99 (1H, m, 7-H), 2.04 (3H, s, 13-H), 2.16 (1H, d, *J* = 18.1 Hz, 2-H), 2.59 (1H, dd, *J* = 4.5, 18.2 Hz, 2-H), 3.66 (1H, qui, *J* = 5.7 Hz, 9-H), 5.83 (1H, s, 4-H). δ_C_ (100 MHz; CD_3_OD) 21.8 (C-13), 23.7 (C-10), 24.0 (C-12), 24.5 (C-11), 35.3 (C-8), 35.7 (C-7), 43.0 (C-1), 51.1 (C-2), 69.3 (C-9), 79.2 (C-6), 126.6 (C-4), 171.8 (C-5), 200.8 (C-3). HRMS (ESI+): Found (MNa^+^): 249.1457 C_13_H_22_NaO_3_ requires 249.1461. IR: ν_max_(film)/cm^−1^; 3405 (O-H), 2965 (C-H), 2927 (C-H), 2878 (C-H), 2530 (O-H), 1647 (C=O), 1417 (C=C). Other diastereomer: δ_H_ (400 MHz; CD_3_OD) 1.02 (3H, s, 11-H), 1.10 (3H, s, 12-H), 1.22 (3H, d, *J* = 6.0 Hz, 10-H), 1.40–1.48 (1H, m, 8-H), 1.68–1.72 (1H, m, 8-H), 1.75–1.83 (1H, m, 7-H), 1.92–1.99 (1H, m, 7-H), 2.04 (3H, s, 13-H), 2.16 (1H, d, *J* = 18.1 Hz, 2-H), 2.59 (1H, dd, *J* = 4.5, 18.2 Hz, 2-H), 3.76–3.78 (1H, m, 9-H), 5.88 (1H, s, 4-H). δ_C_ (100 MHz; CD_3_OD) 21.7 (C-13), 23.5 (C-10), 24.0 (C-12), 24.6 (C-11), 35.2 (C-8), 35.6 (C-7), 42.9 (C-1), 51.1 (C-2), 68.9 (C-9), 79.1 (C-6), 126.6 (C-4), 171.7 (C-5), 200.9 (C-3). HRMS (ESI+): Found (MNa^+^): 249.1457 C_13_H_22_NaO_3_ requires 249.1461. IR: ν_max_(film)/cm^−1^; 3405 (O-H), 2965 (C-H), 2927 (C-H), 2878 (C-H), 2530 (O-H), 1647 (C=O), 1417 (C=C). The spectroscopic data were in agreement with the literature values [25,29].

(*R*)-(But-3-yn-2-yloxy)(*tert*-butyl)diphenylsilane **(*R*)-13**: To a stirred solution of (*R*)-(+)-3-butyn-2-ol (300 mg, 4.28 mmol) in DMF (15 mL) under an nitrogen atmosphere, imidazole (870 mg, 12.8 mmol) and TBDPSCl (1.66 mL, 6.40 mmol) was added at 0 °C, and was stirred for at room temperature for 4 h. The reaction mixture was quenched with sat. aq. NaHCO_3_ (7 mL), and was extracted with ethyl acetate (3 × 15 mL), the combined organic extracts were washed with water (3 × 15 mL), dried over anhydrous MgSO_4_ and the solvent was removed in vacuo. The crude product was purified by flash chromatography (9:1 hexanes, ethyl acetate) to yield the *title compound* **(*R*)-13** (980 mg, 99%) as a colourless oil. R_F_ (9:1 hexanes, ethyl acetate) = 0.72. δ_H_ (400 MHz; CDCl_3_) 1.09 (9H, s, Si(CH_3_)), 1.40 (3H, d, *J* = 6.5 Hz, 1-CH_3_), 2.34 (1H, d, *J* = 2.5 Hz, 4-H), 4.47 (1H, qd, *J* = 6.5, 2.0 Hz, 2-H), 7.36–7.46 (6H, m, Ar-H), 7.60–7.81 (4H, m, Ar-H). ${\left[\alpha \right]}_{D}^{20}=$ +84.4° (*c* = 1.00, MeOH). The spectroscopic data were in agreement with the literature values [34].

(2*R*,3*R*)-2,3,7,9,9-Pentamethyl-1,4-dioxaspiro [4.5]dec-6-en-8-one **(*R*,*R*)-14**: To a stirred solution of 4-oxoisophorone **7** (300 mg, 1.97 mmol) in toluene (8 mL), 2*R*,3*R*-(+)-2,3-butanediol (236 mg, 2.62 mmol) and *p*-toluenesulfonic acid (8.15 mg, 0.0473 mmol) was added. The reaction mixture was heated at reflux for 24 h with a Dean-Stark trap for the removal of excess water. The reaction mixture was cooled and quenched with sat. aq. NaHCO_3_ (8 mL), and then extracted with diethyl ether (3 × 8 mL), dried over anhydrous MgSO_4_ and the solvent was removed in vacuo. The crude product was purified by flash chromatography (14:1 petroleum ether, ethyl acetate) to yield the *title compound* **(*R*,*R*)-14** (280 mg, 93%) as a pale yellow oil. R_F_ (3:1 petroleum ether, ethyl acetate) = 0.75. δ_H_ (400 MHz; CDCl_3_) 1.17 (3H, s, 11-H), 1.22 (3H, s, 12-H), 1.27–1.30 (6H, m, 14 and 15-H), 1.79 (3H, d, *J* = 1.5 Hz, 13-H), 2.03–2.07 (1H, dd, *J* = 14.0, 1.5 Hz, 1H, 10-H), 2.10–2.13 (1H, d, *J* = 14.0 Hz, 10-H), 3.61–3.71 (2H, m, 2 and 3-H), 6.33 (1H, t, *J* = 1.3 Hz, 6-H). ${\left[\alpha \right]}_{D}^{20}=$ −16.1 (*c* = 0.97, MeOH). The spectroscopic data were in agreement with the literature values [35].

(2*R*,3*R*,8*R*)-8-((*R*)-3′((*tert*-Butyldiphenylsilyl)oxy)but-1′-yn-1′-yl-2,3,7,9,9-pentamethyl-1,4-dioxaspiro [4.5]dec-6-en-8-ol **(*R*,*R*)-15**: To a stirred solution of **(*R*)-13** (288 mg, 0.89 mmol) in THF (8 mL) under an nitrogen atmosphere, and *^n^*BuLi (2 M in hexanes, 0.45 mL, 0.89 mmol) was added at −78 °C. The resultant mixture was stirred at −78 °C for 20 min and a solution of **(*R,R*)-14** (100 mg, 0.45 mmol) in THF (4 mL) was added dropwise. The mixture was further stirred under a nitrogen atmosphere at room temperature overnight. The reaction was quenched with sat. aq. NH_4_Cl (6 mL) and was extracted with diethyl ether (3 × 10 mL), the combined organic extracts were washed with water (6 mL), brine (6 mL) and dried over anhydrous MgSO_4_ and the solvent was removed in vacuo. The crude product was recrystallised from petroleum ether to yield the *title compound* **(*R*,*R*)-15** (112 mg, 47%) as a white solid. Melting point: 95–99 °C. δ_H_ (400 MHz; CDCl_3_) 1.02 (3H, s, 11-H), 1.06 (12H, br s, 12-H and SiC(CH_3_)), 1.22 (6H, t, *J* = 5.2 Hz, 16 and 17-H), 1.41 (3H, d, *J* = 6.5 Hz, 10-H), 1.70 (3H, d, *J* = 1.35 Hz, 13-H), 1.76 (1H, d, *J* = 14.1, 2-H), 1.93 (1H, d, *J* = 14.1, 2-H), 1.86 (2H, dd, *J* = 59.6, 14.2 Hz, 2-H), 3.50–3.60 (2H, m, 14 and 15-H), 4.52 (1H, q, *J* = 6.5 Hz, 9-H), 5.30 (1H, s, 4-H), 7.36–7.46 (6H, m, Ar-H), 7.70 (2H, dd, *J* = 8.0, 2.0 Hz, Ar-H), 7.76 (2H, dd, *J* = 8.0, 2.0 Hz, Ar-H). δ_C_ (100 MHz; CDCl_3_) 14.3 (C-13), 18.9 (C-16 and 17), 22.3 (C-11 and 12), 25.5 (C-10), 26.9 (SiC(Ph)_2_C(CH_3_)_3_), 39.4 (SiC(Ph)_2_C(CH_3_)_3_), 45.6 (C-2), 60.1 (C-9), 74.3 (C-7), 78.0 (C-14 and 15), 84.1 (C-8), 88.1 (C-6), 104.1 (C-3), 124.8 (C-4), 136.0 (Si(CH_3_)), 140.4 (C-5). HRMS (ESI+): Found (MNa^+^): 555.2898 C_33_H_44_NaO_4_Si requires 555.2901.

(*R*)-6-Hydroxy-6-((*R*)-9-hydroxybut-7-yn-7-yl)-1,1,5-trimethylcyclohex-4-en-3-one **(*R*,*R*)-17**: To a stirred solution of **(*R*,*R*)-15** (703 mg, 1.32 mmol) in THF (40 mL) under a nitrogen atmosphere, 2M HCl (6 mL) was added and the mixture stirred under an nitrogen atmosphere at room temperature overnight. The solvent was removed in vacuo and the remaining residue was extracted with ethyl acetate (3 × 40 mL). The combined organic extracts were washed brine (40 mL) and dried over anhydrous MgSO_4_ and the solvent was removed in vacuo. This crude intermediate **(*R*,*R*)-16** was dissolved in THF (40 mL) under a nitrogen atmosphere, TBAF (0.73 mL, 2.52 mmol) was added at 0 °C. The resultant mixture was stirred under a nitrogen atmosphere at room temperature overnight. The reaction was quenched with water (40 mL), and the solvent was removed in vacuo, and was extracted with ethyl acetate (3 × 40 mL), the combined organic extracts were washed brine (40 mL) and dried over anhydrous MgSO_4_ and the solvent was removed in vacuo. The crude product was purified by flash chromatography (1:3 petroleum ether, ethyl acetate) to yield the *title compound* **(*R*,*R*)-17** (270 mg, 91%) as a colourless oil. N.B. compound **17** was found to be very volatile and prone to evaporation if placed under strong vacuum. R_F_ (1:1 petroleum ether, ethyl acetate) = 0.23. δ_H_ (400 MHz; CDCl_3_) 1.09 (3H, s, 11-H), 1.19 (3H, s, 12-H), 1.47 (3H, d, *J* = 6.5 Hz, 10-H), 2.10 (3H, d, *J* = 1.35 Hz, 13-H), 2.38 (1H, d, *J* = 16.5 Hz, 2-H), 2.51 (1H, d, *J* = 16.5 Hz, 2-H), 4.58 (1H, q, *J* = 6.6, 9-H), 5.83 (1H, s, 4-H). δ_C_ (100 MHz; CDCl_3_) 19.8 (C-12), 22.8 (C-11), 24.4 (C-10), 25.2 (C-13), 29.7 (C-1), 49.4 (C-2), 58.4 (C-9), 74.5 (C-7), 82.8 (C-5), 89.4 (C-8), 126.3 (C-4), 160.4 (C-5), 198.4 (C-3). HRMS (ESI+): Found (MNa^+^): 245.2712 C_13_H_18_NaO_3_ requires 245.2712. ${\left[\alpha \right]}_{D}^{19.8}$ = −138.79° (*c* = 0.66, MeOH). IR: ν_max_(film)/cm^−1^; 3456, 3025, 2864, 1396, 1086.

(6*R*,9*R*)-Blumenol B **(*R,R*)-1**: To a stirred solution of **(*R*,*R*)-17** (14 mg, 0.0629 mmol) in MeOH (5 mL), Pd/BaSO_4_ (1.4 mg, 20% w/w) was added, and the resultant mixture was stirred under a hydrogen atmosphere overnight. The mixture was filtered through Celite^®^, washed with MeOH and the solvent was removed in vacuo. The crude product was purified by flash chromatography (1:2 petroleum ether, ethyl acetate) to yield the *title compound* **(*R,R*)-1** (8 mg, 57%) as a colourless oil. R_F_ (1:3 petroleum ether, ethyl acetate) = 0.26. δ_H_ (400 MHz; CD_3_OD) 1.02 (3H, s, 11-H), 1.09 (3H, s, 12-H), 1.16 (3H, d, *J* = 6.2 Hz, 10-H), 1.40 (1H, dddd, *J* = 12.7, 11.5, 8.05, 5.12 Hz, 8-H), 1.68 (1H, tt, *J* = 12.7, 4.5 Hz, 8-H), 1.79 (1H, td, *J* = 12.7, 4.0 Hz, 7-H), 1.98 (1H, td, *J* = 12.7, 5.3 Hz, 7-H), 2.04 (3H, d, *J* = 1.49 Hz, 13-H), 2.16 (1H, dd, *J* = 18.0, 1.02 Hz, 2-H), 2.59 (1H, d, *J* = 18.1 Hz, 2-H), 3.61–3.69 (1H, m, 9-H), 5.83 (1H, s, 4-H). δ_C_ (100 MHz; CD_3_OD) 21.8 (C-13), 23.7 (C-10), 24.0 (C-11), 24.5 (C-12), 35.3 (C-7), 35.7 (C-8), 42.9 (C-1), 51.5 (C-2), 69.3 (C-9), 79.2 (C-6), 126.6 (C-4), 171.8 (C-5), 200.8 (C-3). HRMS (ESI+): Found (MNa^+^): 249.3115 C_13_H_22_NaO_3_ requires 249.3115. ${\left[\alpha \right]}_{D}^{22}$ = −1.25° (*c* = 0.16, MeOH). IR: ν_max_(film)/cm^−1^; 3469, 3068, 2956, 1410, 1097.

Compounds **d_9_-19**, **d_9_-20** and **d_9_-8** were prepared using the reported method [36].

2,6,6-Tris(methyl-d_3_)cyclohex-2-ene-1,4-dione-d_9_ **d_9_-7:** To a stirred solution of **d_9_-8** (61 mg, 0.297 mmol) in THF (7 mL) under a nitrogen atmosphere, 2M HCl (3.5 mL) was added, and the resultant mixture was stirred under a nitrogen atmosphere at room temperature overnight. The solvent was removed in vacuo and was extracted with ethyl acetate (3 × 7 mL) and the combined organic extracts were washed with brine (7 mL), and dried over anhydrous MgSO_4_, and the solvent was removed in vacuo. The crude product was purified by flash chromatography (4:1 petroleum ether, ethyl acetate) to yield the *title compound* **d_9_-7** (48 mg, quant.) as a colourless oil. **R_F_** (3:1 petroleum ether, ethyl acetate) = 0.61. **δ_H_** (400 MHz; CDCl_3_) 2.70 (2H, s, 5-H), 6.55 (1H, s, 3-H). **δ_C_** (100 MHz; CDCl_3_) 44.9 (C-6), 51.8 (C-5), 137.2 (C-3), 149.0 (C-2), 197.9 (C-4), 203.7 (C-1). **HRMS** (ESI+): Found (MNa^+^): 184.2413 C_9_H_3_D_9_NaO_2_ requires 184.2412. **IR**: ν_max_(film)/cm^−1^; 3049, 2986, 2912, 1768, 1087.

(2*R*,3*R*)-2,3-dimethyl-7,9,9-tris(methyl-d_3_)-1,4-dioxaspiro [4.5]dec-6-en-8-one-d_9_ **d_9_-(*R*,*R*)-14**: To a stirred solution of **d_9_-7** (1 g, 6.20 mmol) in toluene (15 mL), a solution of 2*S*,3*S*-(+)-2,3-butanediol (0.68 mL, 7.44 mmol) and *p*-toluenesulfonic acid (37 mg, 0.217 mmol) was added, and the resultant mixture was heated at reflux for 24 h with a Dean–Stark trap for the removal of excess water. The reaction mixture was cooled and quenched with sat. aq. NaHCO_3_ (15 mL), and was extracted with diethyl ether (3 × 8 mL), dried over anhydrous MgSO_4_ and the solvent removed in vacuo. The crude product was purified by flash chromatography (14:1 petroleum ether, ethyl acetate) to yield the *title compound* **d_9_-(*R*,*R*)-14** (1.24 g, 89%) as a white solid. R_F_ (9:1 petroleum ether, ethyl acetate) = 0.5. Melting point: 70–76 °C. δ_H_ (400 MHz; CDCl_3_) 1.28 (6H, t, *J* = 5.45 Hz, 11 and 12-H), 2.02–2.13 (2H, m, 10-H), 3.61–3.71 (2H, m, 2 and 3-H), 6.32 (1H, s, 6-H). δ_C_ (100 MHz; CDCl_3_) 16.7 and 16.9 (C-11 and 12), 47.5 (C-10), 78.5 (C-2 and 3), 103.0 (C-5), 141.5 (C-6), 204.6 (C-8). HRMS (ESI+): Found (MNa^+^): 256.3423 C_13_H_11_D_9_NaO_3_ requires 256.3428. ${\left[\alpha \right]}_{D}^{20.5}=$ + 5.50 (*c* = 0.4, CHCl_3_). IR: ν_max_(film)/cm^−1^; 3027, 2971, 1695, 1521, 1063.

(6*R*,14*R,*15*R*)-6-((*R*)-9-((*tert*-Butyldiphenylsilyl)oxy)but-7-yn-7-yl)-14,15-dimethyl-1,1,5-tris(methyl-d_3_)-18,19-dioxaspiro [3.19]dec-4-en-6-ol-d_9_ **d_9_-(*R*,*R*)-15**: To a stirred solution of **(*R*)-13** (1.62 g, 5.04 mmol) in THF (30 mL) under a nitrogen atmosphere, *^n^*BuLi (2 M in hexanes, 2.52 mL, 5.04 mmol) was added at −78 °C. The reaction mixture was further stirred at −78 °C for 20 min and **d_9_-(*R*,*R*)-14** (588 mg, 2.52 mmol) in THF (30 mL) was added dropwise. The resultant mixture was then further stirred at room temperature under a nitrogen atmosphere overnight. The reaction was quenched with sat. aq. NH_4_Cl (30 mL) and was extracted with ethyl acetate (3 × 30 mL), the combined organic extracts were washed with water (30 mL), brine (30 mL) and dried over anhydrous MgSO_4_ and the solvent was removed in vacuo. The crude product was purified by flash chromatography (9:1 petroleum ether, ethyl acetate) and recrystallised from petroleum ether to yield the *title compound* **d_9_-(*R*,*R*)-15** (1.98 g, 51%) as a white solid. R_F_ (4:1 petroleum ether, ethyl acetate) = 0.41. Melting point: 111–115 °C. δ_H_ (400 MHz; CDCl_3_) 1.05 (9H, s, Si(CH_3_)), 1.20–1.23 (6H, t, *J* = 4.8 Hz, 16 and 17-H), 1.41 (3H, d, *J* = 6.5 Hz, 10-H), 1.52 (1H, br s, OH), 1.74 (1H, d, *J* = 14.2 Hz, 2-H), 1.92 (1H, d, *J* = 14.1 Hz, 2-H), 3.50–3.60 (2H, m, 14 and 15-H), 4.52 (1H, q, *J* = 6.4 Hz, 9-H), 5.28 (1H, s, 4-H), 7.34–7.44 (6H, m, Ar-H), 7.68–7.75 (4H, m, Ar-H). δ_C_ (100 MHz; CDCl_3_) 16.8 (C-16), 16.9 (C-17), 19.2 (2 x CD_3_), 25.3 (C-10), 26.7 ((-OSi(Ph-C)C(CH_3_)_3_)), 26.9 and 39.0 (-OSi(Ph-C)C(CH_3_)_3_)), 45.5 (C-2), 60.1 (C-9), 74.3 (C-7), 77.9 and 78.0 (C-14 and 15), 84.1 (C-8), 88.1 (C-6), 104.1 (C-3), 124.8 (C-4), 127.6–136.0 (-OSi(Ph-C)C(CH_3_)_3_)), 140.3 (C-5). HRMS (ESI+): Found (MNa^+^): 564.8435 C_33_H_35_D_9_NaO_4_Si requires 564.8435. ${\left[\alpha \right]}_{D}^{21.5}$ = + 2.27° (*c* = 0.44, CHCl_3_). IR: ν_max_ (film)/cm^−1^; 3574, 3058, 2871, 2133, 1739, 1524, 1088.

(*R*)-6-((*R*))-9-((*tert*-Butyldiphenylsilyl)oxy)but-7-yn-7-yl)-6-hydroxy-1,1,5-tris(methyl-d_3_)cyclohex-4-en-3-one-d_9_ **d_9_-(*R*,*R*)-16**: To a stirred solution of **d_9_-(*R*,*R*)-15** (1.98 g, 3.71 mmol) in THF (40 mL) under a nitrogen atmosphere, 2M HCl (10 mL) was added, and the reaction mixture was stirred under anitrogen atmosphere at room temperature overnight. The solvent was removed in vacuo and was extracted with ethyl acetate (3 × 40 mL); the combined organic extracts were washed with brine (40 mL), dried over anhydrous MgSO_4_ and the solvent was removed in vacuo. The crude product was purified by flash chromatography (3:1 petroleum ether, ethyl acetate) to yield the *title compound* **d_9_-(*R*,*R*)-16** (1.60 g, 94%) as white crystals. R_F_ (3:1 petroleum ether, ethyl acetate) = 0.51. Melting point: 105–107 °C. δ_H_ (400 MHz; CDCl_3_) 1.05 (9H, s, Si(CH_3_)), 1.46 (3H, d, *J* = 6.5 Hz, 10-H), 2.25 (1H, d, *J* = 16.5 Hz, 2-H), 2.34 (1H, d, *J* = 16.5 Hz, 2-H), 4.57 (1H, q, *J* = 6.5 Hz, 9-H), 5.73 (1H, s, 4-H), 7.36–7.46 (6H, m, Ar-H), 7.70 (4H, dt, *J* = 20.4, 6.1, 1.5 Hz, Ar-H). δ_C_ (100 MHz; CDCl_3_) 19.2 (C-1), 25.2 (C-10), 26.8 ((-OSi(Ph-C)C(CH_3_)_3_)), 41.2 (C-2), 59.9 (C-9), 74.2 (C-6), 82.4 (C-8), 89.7 (C-7), 126.1 (C-4), 127.7 and 127.9 (-OSi(Ph-C)C(CH_3_)_3_)), 130.0 (-OSi(Ph-C)C(CH_3_)_3_)), 135.8 and 136.0 (-OSi(Ph-C)C(CH_3_)_3_)), 160.0 (C-5), 198.3 (C-3). HRMS (ESI+): Found (MNa^+^): 492.2878 C_29_H_27_D_9_NaO_3_Si requires 492.2891. ${\left[\alpha \right]}_{D}^{21.7}$ = −2.35° (*c* = 0.34, CHCl_3_). IR: ν_max_(film)/cm^−1^; 3369, 3056, 2863, 1786, 1121.

(*R*)-4-Hydroxy-4-((*R*)-3′-hydroxybut-1-yn-1-yl)-3,5,5-tris(methyl-d_3_)cyclohex-2-en-1-one-d_9_ **d_9_-(*R*,*R*)-17**: To a stirred solution of **d_9_-(*R*,*R*)-16** (69 mg, 0.15 mmol) in THF (7 mL) under a nitrogen atmosphere, TBAF (0.09 mL, 0.30 mmol) was added at 0 °C, and the resultant mixture was stirred at room temperature under a nitrogen atmosphere overnight. The reaction was quenched with water (7 mL), the solvent was removed in vacuo and extracted with ethyl acetate (3 × 7 mL), the combined organic extracts were washed with brine (7 mL) and dried over anhydrous MgSO_4_ and the solvent was removed in vacuo. The crude product was purified by flash chromatography (1:2 petroleum ether, ethyl acetate) to yield the *title compound* **d_9_-(*R*,*R*)-17** (35 mg, quant.) as a colourless oil. N.B. compound **17** was found to be very volatile and was prone to evaporation if placed under a strong vacuum. R_F_ (1:1 petroleum ether, ethyl acetate) = 0.26. δ_H_ (400 MHz; CDCl_3_) 1.46 (3H, d, *J* = 6.5 Hz, 10-H), 2.37 (1H, d, *J* = 16.4 Hz, 2-H), 2.49 (1H, d, *J* = 16.4 Hz, 2-H), 2.83 (OH), 4.58 (1H, q, *J* = 6.6 Hz, 9-H), 5.83 (1H, s, 4-H). δ_C_ (100 MHz; CDCl_3_) 24.4 (C-10), 49.2 (C-2), 58.4 (C-9), 74.4 (C-6), 82.7 (C-7), 89.3 (C-8), 126.2 (C-4), 198.5 (C-3). HRMS (ESI+): Found (MNa^+^): 254.1704 C_13_H_9_D_9_NaO_3_ requires 254.1713. ${\left[\alpha \right]}_{D}^{20.1}$ = −150.51° (*c* = 0.78, MeOH). IR: ν_max_(film)/cm^−1^; 3594, 2861, 2236, 1811, 1067.

(6*R,*9*R*)-Blumenol B-d_9_ **d_9_-(*R,R*)-1**: To a stirred solution of **d_9_-(*R*,*R*)-17** (54 mg, 0.233 mmol) in MeOH (7 mL), Pd/BaSO_4_ (10 mg, 20% w/w) was added, and was stirred under a hydrogen atmosphere for 6 h. The mixture was filtered through Celite^®^, washed with MeOH and the solvent was removed in vacuo. The crude product was purified by flash chromatography (1:3 petroleum ether, ethyl acetate) to yield the *title compound* **d_9_-(*R*,*R*)-1** as (24 mg, 44%) as a colourless oil. R_F_ (1:3 petroleum ether, ethyl acetate) = 0.24. δ_H_ (400 MHz; CD_3_OD) 1.17 (3H, d, *J* = 6.2 Hz, 10-H), 1.34–1.44 (1H, m, 8-H), 1.64–1.82 (2H, m, 8-H), 1.94–2.02 (1H, m, 7-H), 2.15 (1H, dd, *J* = 18.1, 0.98 Hz, 2-H), 2.59 (1H, d, *J* = 18.1 Hz, 2-H), 3.62–3.69 (1H, m, 9-H), 5.83 (1H, s, 4-H). δ_C_ (100 MHz; CD_3_OD) 23.7 (C-10), 35.3 (C-7), 35.7 (C-8), 50.9 (C-2), 69.4 (C-9), 79.2 (C-6), 126.6 (C-4), 171.7 (C-5), 200.9 (C-3). HRMS (ESI+): Found (MNa^+^): 258.2018 C_13_H_13_D_9_NaO_3_ requires 258.2026. ${\left[\alpha \right]}_{D}^{27.2}$ = −2.272° (*c* = 0.22, CHCl_3_). IR: ν_max_(film)/cm^−1^; 3412.1 (broad OH), 2850.9, 2917.2 and 2961.0 (C-H), 1650.9 and 1729.3 (C=O), 1275.4 (C-O).

## 4. Conclusions

In summary, we developed a six-step enantioselective route for the synthesis of (*R*,*R*) blumenol B **1**. The synthesis is high-yielding, efficient, and significantly shorter than previously reported methods. Furthermore the method can be applied to the synthesis of d_9_-labelled (*R*,*R*)-blumenol B **d_9_-(*R,R*)-1**. The enantioselective synthesis involving the use of two readily available chiral reagents allowed for the selective formation and easy separation of respective stereoisomers.

## Supplementary Materials

The following supporting information can be downloaded at: https://www.mdpi.com/article/10.3390/molecules27217294/s1.

## Sample Availability

Samples of the compounds are not available from the authors.
