# Supplementary material for: Enantioselective Total Synthesis of (R,R)-Blumenol B and d9-(R,R)-Blumenol B
Source: Molecules. 2022 Oct 27;27(21):7294. doi: 10.3390/molecules27217294 (PMC9655556; doi:10.3390/molecules27217294)
Supplement: Supplementary file 1 [file molecules-27-07294-s001.zip › molecules-1949371-supplementary.pdf]

Supporting Information

Enantioselective total synthesis of (*R*,*R*)-blumenol B and d9-(*R*,*R*)-blumenol B.

Shi Min Tan, Jin Wang, Shaun W. P. Rees, Rebecca Jelley, Bruno Fedrizzi* and David Barker*.

*b.fedrizzi@auckland.ac.nz (B. Fedrizzi), d.barker@auckland.ac.nz (D. Barker).

Supplementary Tables: S2

NMR Spectra of Novel Synthesized Compounds: S7

X-Ray diffraction (XRD) analysis: S14

Supplementary Tables:

Table S1. ^{1}H NMR data of natural and synthetic (±)-**1** in CD_3OD.

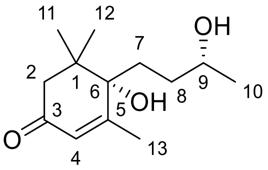

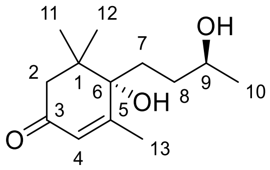

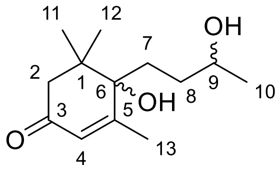

	(*S*,*R*)-Blumenol B[29]	(*S*,*S*)-Blumenol B[29]	(±)-Blumenol B (**1**) synthesised Major Diastereomer	(±)-Blumenol B (**1**) synthesised Minor Diastereomer
Position	δ_H mult. (*J* in Hz)	δ_H mult. (*J* in Hz)	δ_H mult. (*J* in Hz)	δ_H mult. (*J* in Hz)
2	2.16 dd (18, 1)	2.16 dd (18, 1)	2.16 d (18.1)	2.16 d (18.1)
	2.58 d (18)	2.59 d (18)	2.60 d (18.1)	2.59 d (18.1)
4	5.83 dq (1, 1)	5.83 dq (1, 1)	5.83 s	5.83 s
7	1.77 ddd (14, 12, 5)	1.79 ddd (14, 12, 4)	1.86−1.92 m	1.86−1.92 m
	1.95 ddd (14, 12, 4)	1.98 ddd (14, 12, 5)	1.92−2.01 m	1.92−2.01 m
8	1.43 dddd (13, 12, 5, 5)	1.40 dddd (13, 12, 8, 5)	1.36−1.47 m	1.36−1.47 m
	1.65 dddd (13, 12, 7, 4)	1.68 dddd (13, 12, 5, 4)	1.64−1.72 m	1.64−1.72 m
9	3.66 dqd (7, 6, 5)	3.65 dqd (8, 6, 5)	3.62−3.69 m	3.62−3.69 m
10	1.15 d (6)	1.16 d (6)	1.17 d (6.2)	1.15 d (6.2)
11	1.02 s	1.02 s	1.02 s	1.02 s
12	1.10 s	1.09 s	1.10 s	1.10 s
13	2.04 d (1)	2.04 d (1)	2.04 br s	2.04 br s

Table S2. ^{13}C NMR data of natural and synthetic (±)-**1** in CD_3OD.

	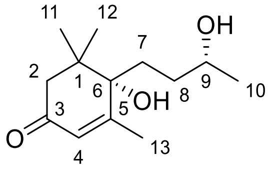	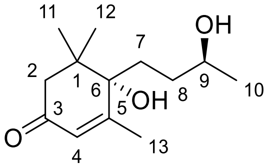	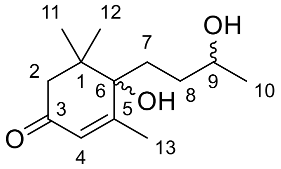	
	(*S*,*R*)-Blumenol B[29]	(*S*,*S*)-Blumenol B[29]	(±)-Blumenol B (**1**) synthesised	
			Major Diastereomer	Minor Diastereomer
Position	δ_C	δ_C	δ_C	δ_C
1	43.0	43.0	43.0	42.9
2	51.2	51.2	51.1	51.1
3	200.9	200.9	200.8	200.9
4	126.7	126.7	126.5	126.6
5	171.7	171.8	171.8	171.7
6	79.2	79.3	79.2	79.1
7	35.3	35.8	35.7	35.7
8	35.4	35.4	35.3	35.2
9	69.0	69.4	69.3	68.9
10	23.5	23.7	24.0	24.0
11	24.7	24.6	23.7	23.7
12	24.1	24.1	23.7	23.7
13	21.8	21.8	21.8	21.7

Table S3. ^{1}H and ^{13}C assignments for synthesized (*R*,*R*)-blumenol B **1** and (*S*,*S*)-blumenol B[29] in CD_3OD.

	(***R***,***R***)-Blumenol B 1		(***S***,***S***)-Blumenol B natural	
Position	δ_H mult (*J* in Hz)	δ_C	δ_H mult (*J* in Hz)	δ_C
1	N/A	42.9	N/A	43.0
2	2.16, dd (18.0, 1.08)<br>2.59, d (18.1)	51.1	2.16, dd (18.0, 1.0)<br>2.59, d (18.0)	51.2
3	N/A	200.8	N/A	200.9
4	5.83, s	126.6	5.83, s	126.7
5	N/A	171.8	N/A	171.8
6	N/A	79.2	N/A	79.3
7	1.40, dddd (12.7, 11.5, 8.1, 5.1)<br>1.68, dddd (13.0, 12.2, 5.0, 3.9)	35.3	1.40, dddd (13, 12, 8, 5)<br>1.68 dddd (13, 12, 5, 4)	35.8
8	1.79, ddd (13.6, 12.9, 3.8)<br>1.97, ddd (27.3, 13.0, 5.2)	35.7	1.79, ddd (14, 12, 4)<br>1.98, ddd (14, 12, 5)	35.4
9	3.61−3.69, m	69.3	3.65, dqd (8, 6, 5)	69.4
10	1.17, d (6.2)	23.7	1.16, d (6)	23.7
11	1.09, s	24.0	1.09, s	24.6
12	1.02, s	24.5	1.02, s	24.1
13	2.04, d (1.4)	21.8	2.04, d (1)	21.8

NMR Spectra of Novel Synthesized Compounds:

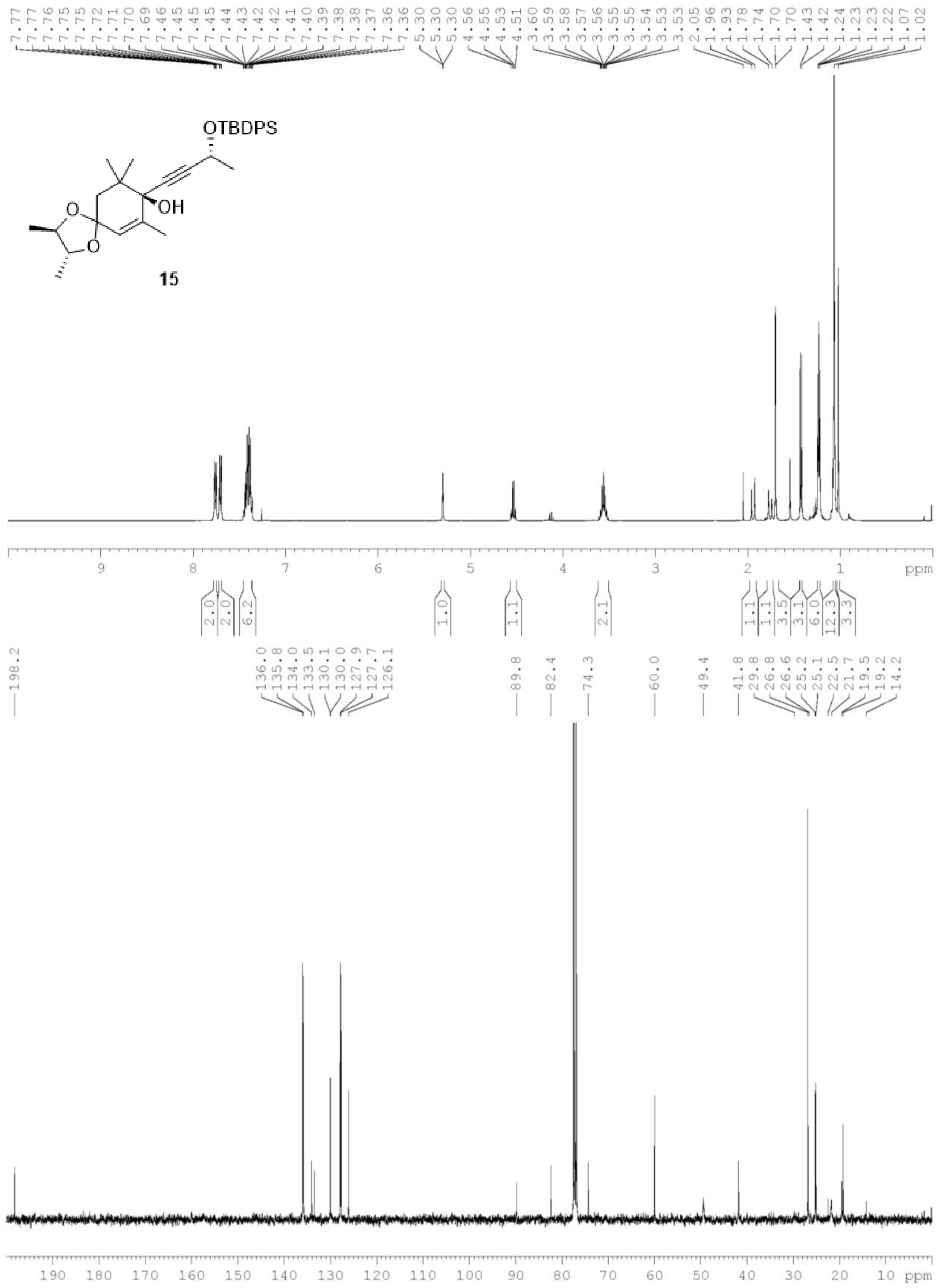

Figure S1: ^{1}HNMR and ^{13}CNMR Spectra of **15**.

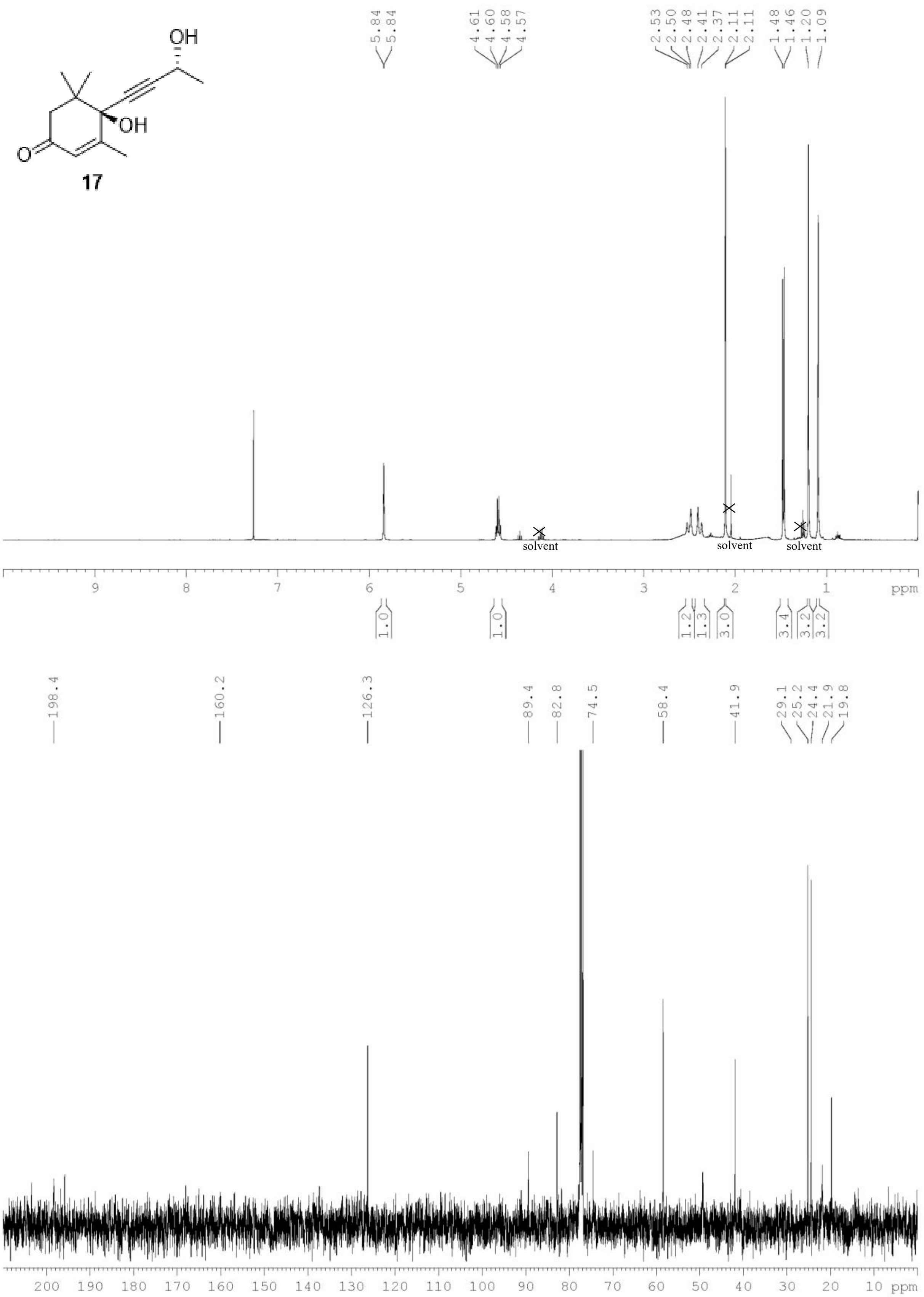

Figure S2: ^{1}HNMR and ^{13}CNMR Spectra of **17**. This compound was found to be very volatile was prone to evaporation if placed under strong vacuum.

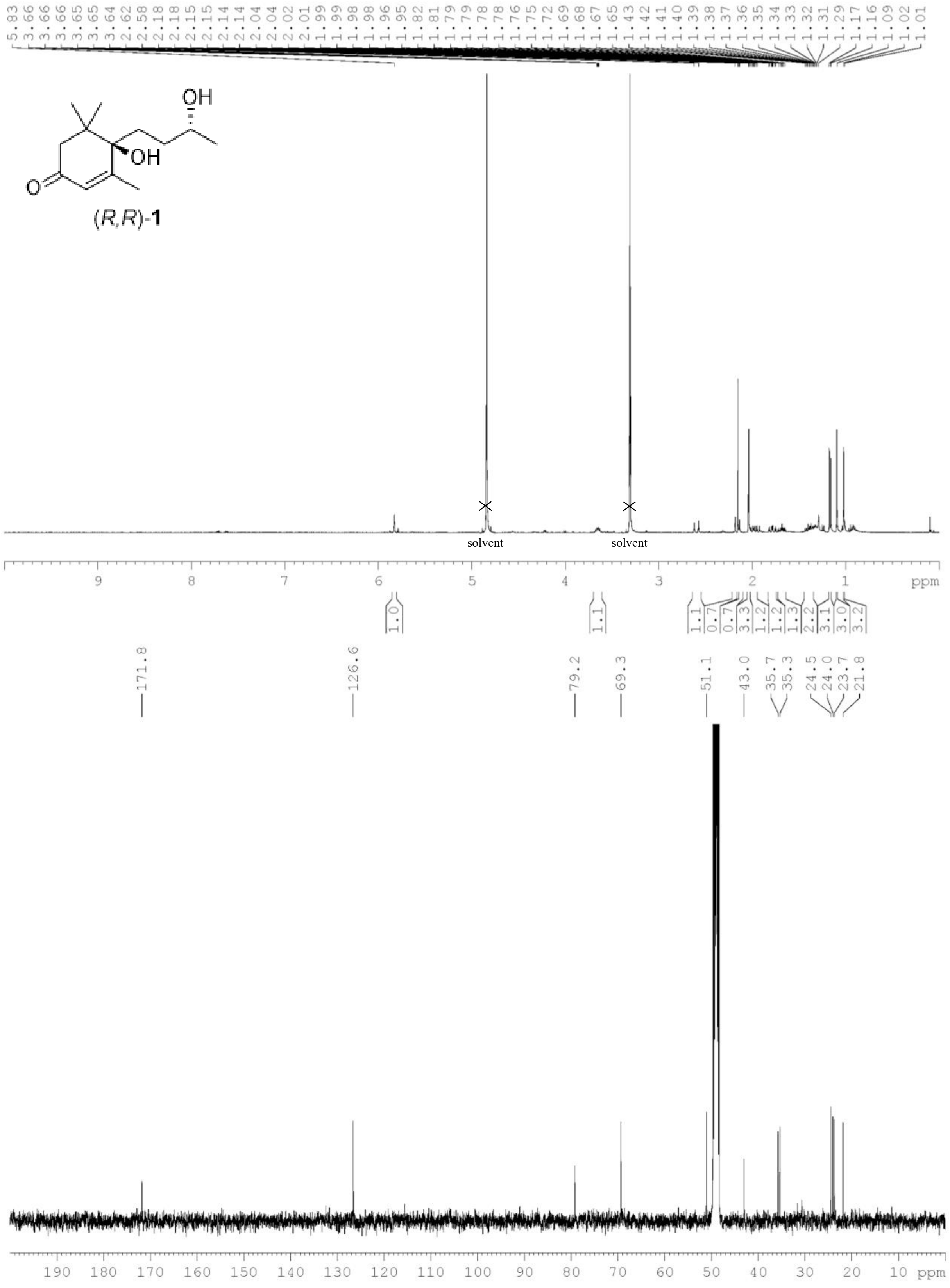

Figure S3: ^{1}HNMR and ^{13}CNMR Spectra of (*R,R*)-**1**.

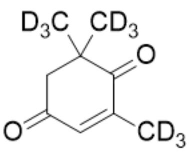

d9-**7**

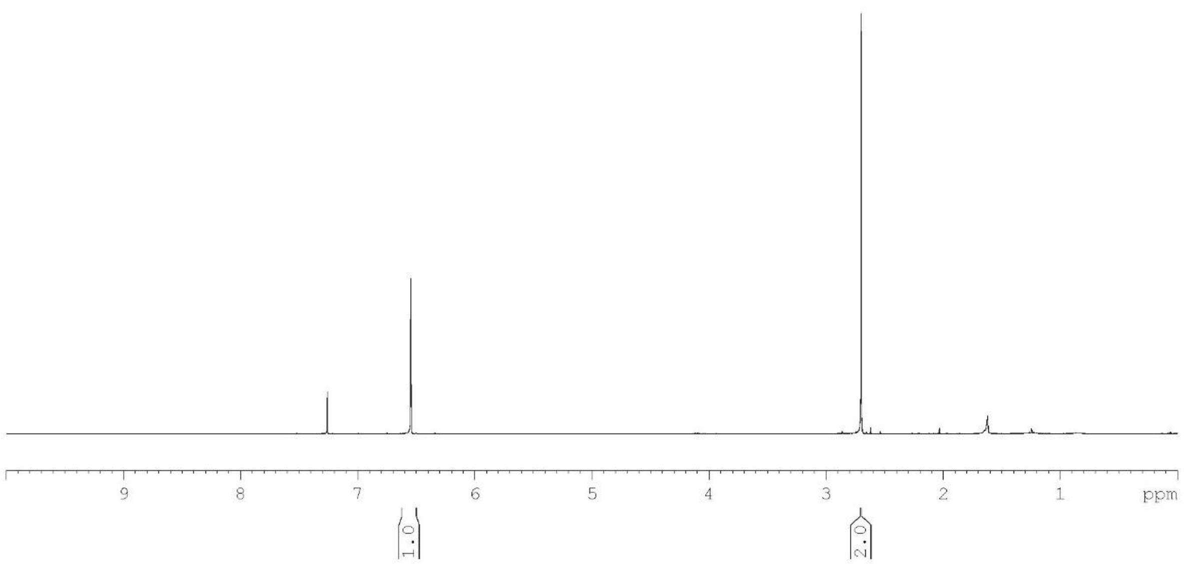

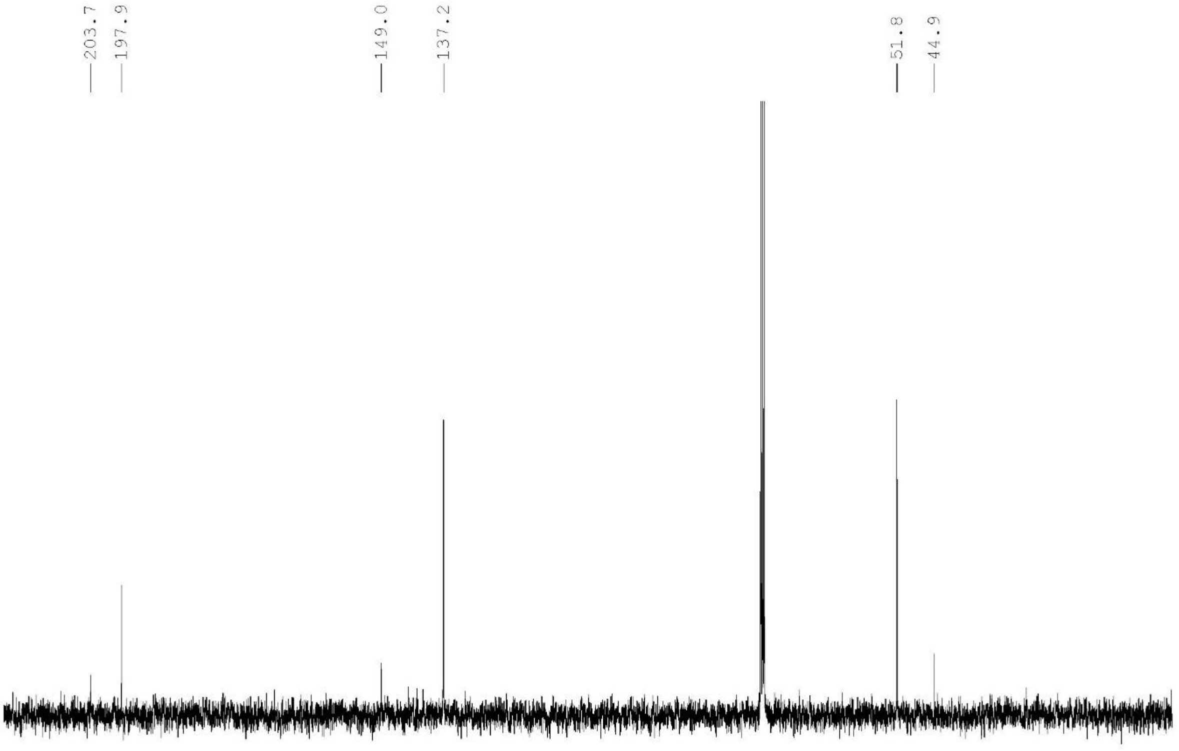

Figure S4: ^{1}HNMR and ^{13}CNMR Spectra of d9-**7**.

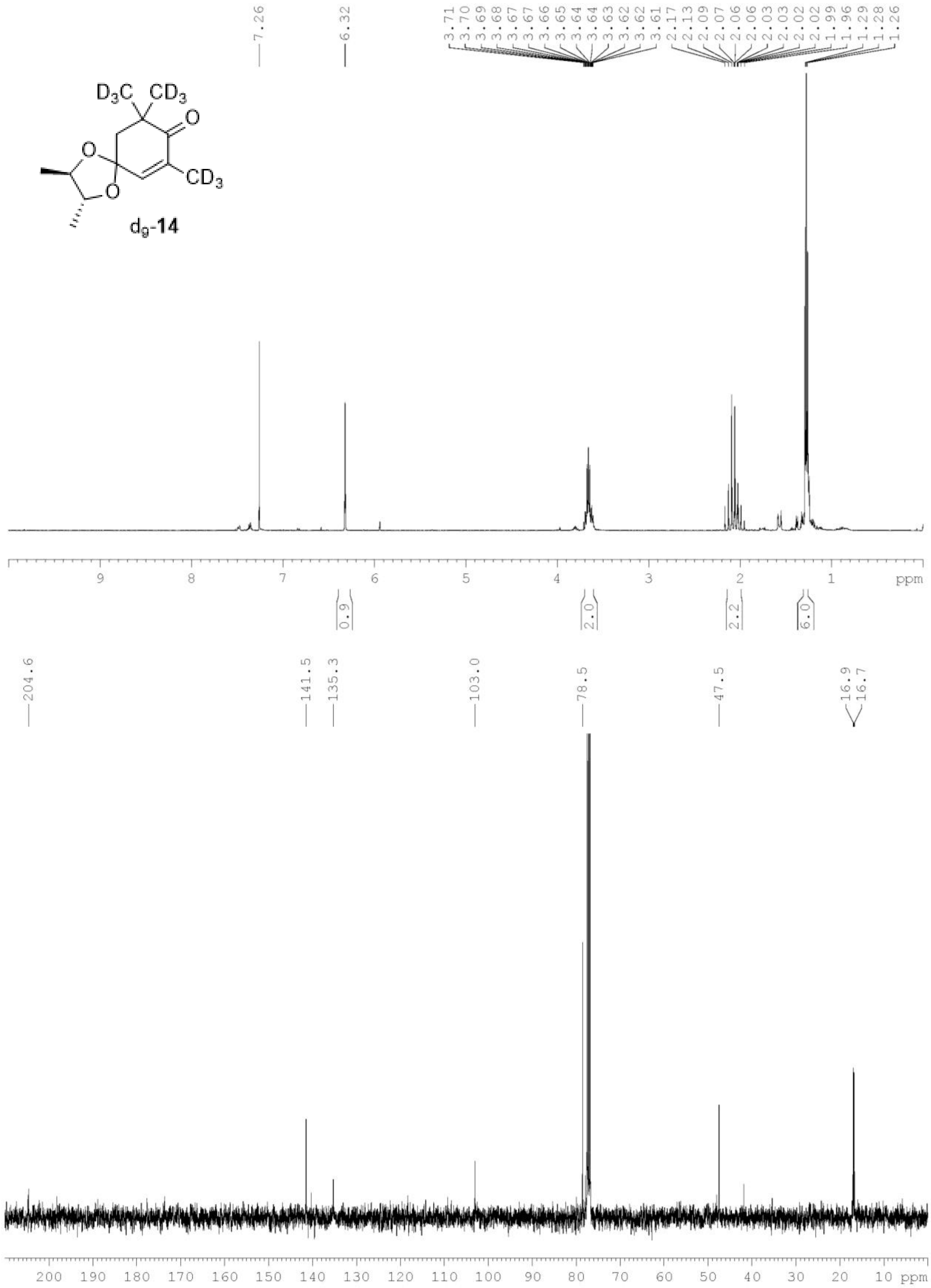

Figure S5: ^{1}HNMR and ^{13}CNMR Spectra of d_9-**14**.

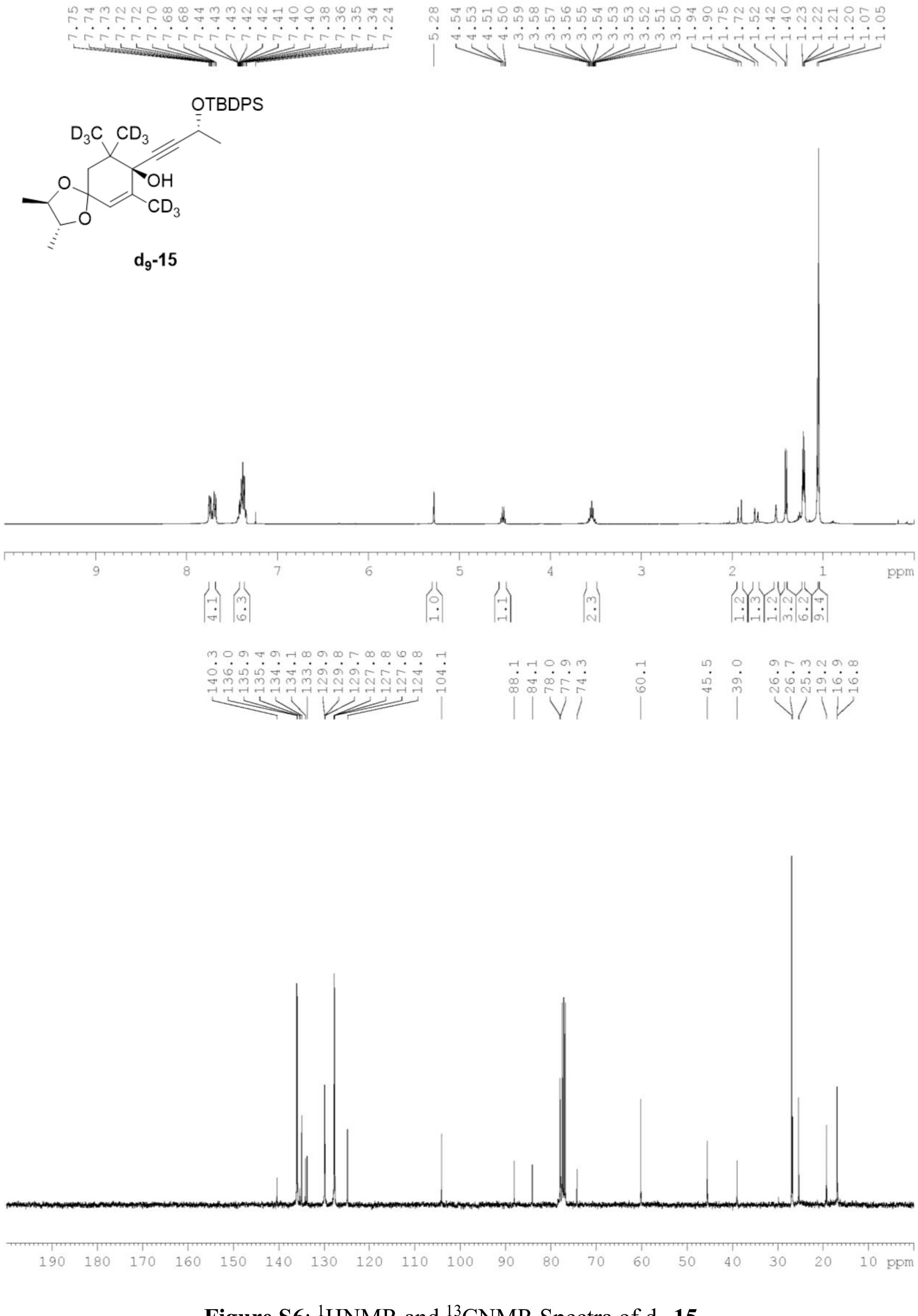

Figure S6: ^{1}HNMR and ^{13}CNMR Spectra of d9-**15.**

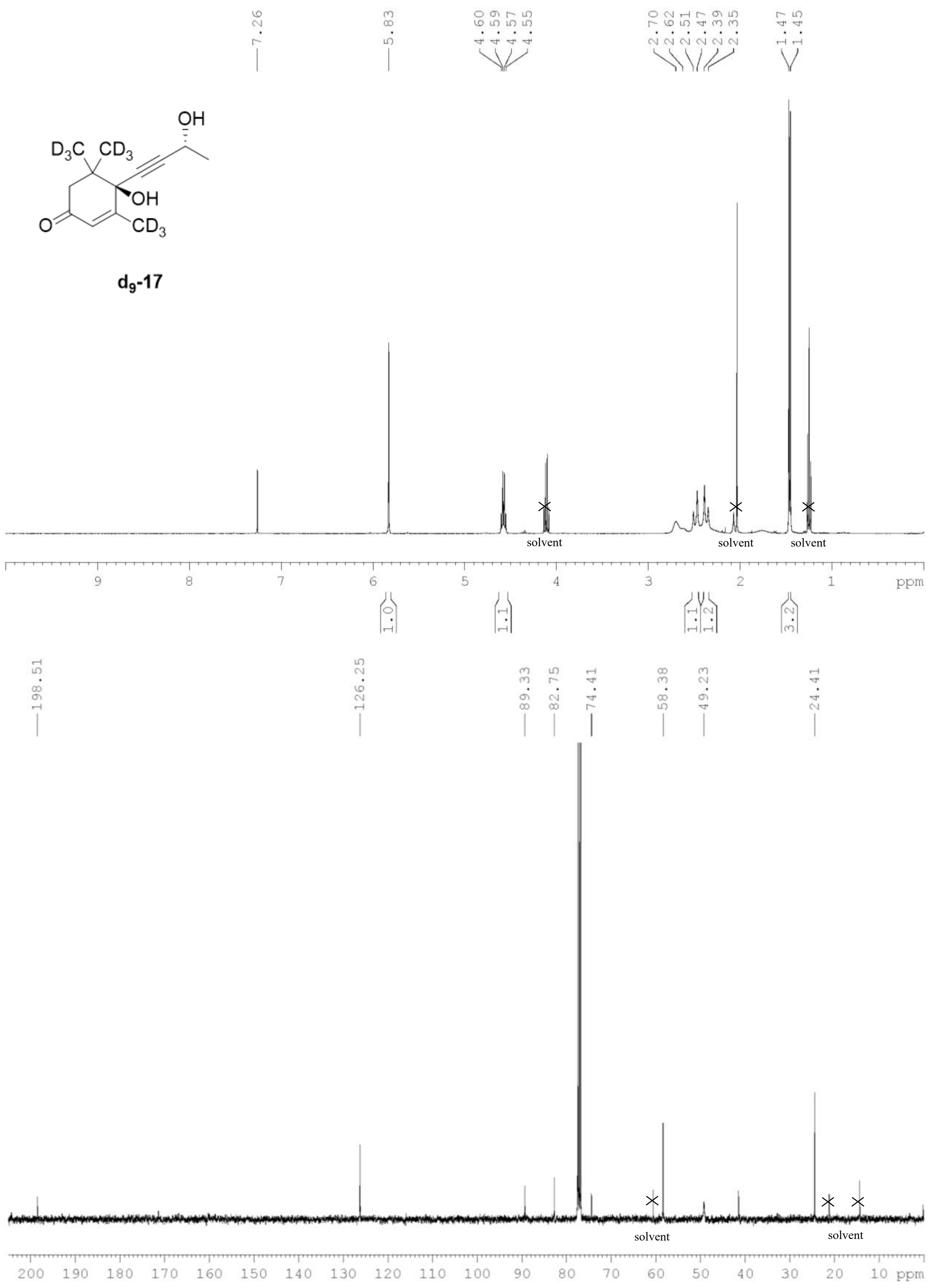

Figure S7: ^{1}HNMR and ^{13}CNMR Spectra of d9-**17.** This compound was found to be very volatile was prone to evaporation if placed under strong vacuum.

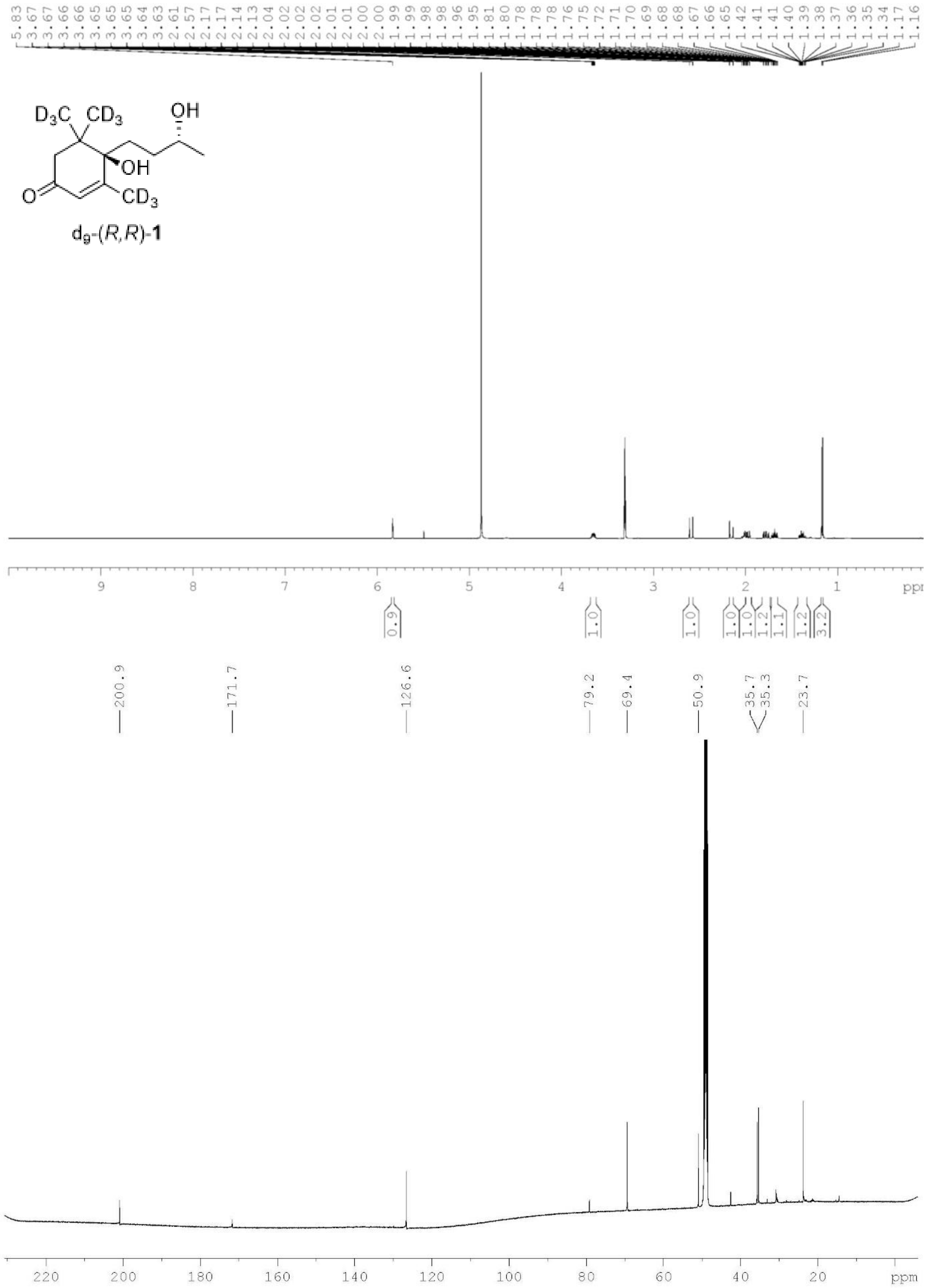

Figure S8: ^{1}HNMR and ^{13}CNMR Spectra of d9-(*R,R*)-**1**.

X-Ray diffraction (XRD) analysis for compound d9-(*R,R*)-16:

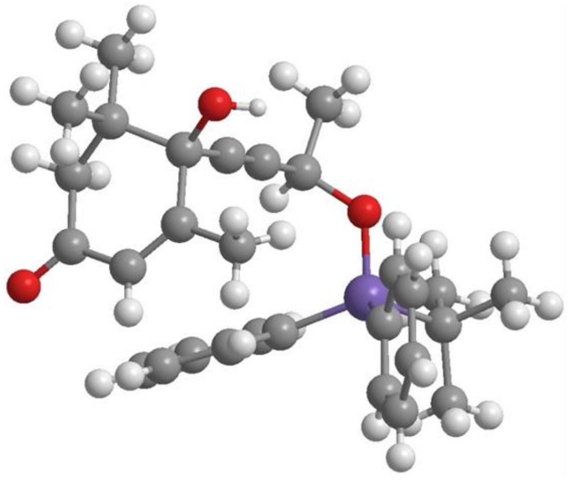

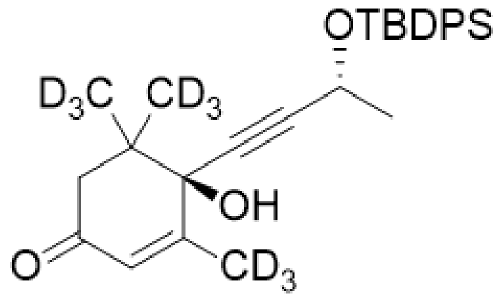

d9-(*R*,*R*)-16

Figure S1: XRD analysis of **d9-*(R*,*R*)-16**.

Experimental

A suitable single crystal of $C_{29}H_{36}O_3Si$ [**d9-*(R*,*R*)-16**] was selected and run on a XtaLAB Synergy, Dualflex, Pilatus 200K diffractometer. The crystal was kept at 100.02(10) K during data collection. Using Olex2 [1], the structure was solved with the ShelXT [2] structure solution program using Intrinsic Phasing and refined with the ShelXL [3] refinement package using Least Squares minimisation.

[1] Dolomanov, O.V., Bourhis, L.J., Gildea, R.J, Howard, J.A.K. & Puschmann, H. (2009), J. Appl. Cryst. 42, 339-341.

[2] Sheldrick, G.M. (2015). Acta Cryst. A71, 3-8.

[3] Sheldrick, G.M. (2015). Acta Cryst. C71, 3-8.

Crystal Data for [**d9-*(R*,*R*)-16**] $C_{29}H_{36}O_3Si$ (M =460.67 g/mol): monoclinic, space group C2 (no. 5), a = 15.0513(3) Å, b = 13.3427(2) Å, c = 13.3314(3) Å, β = 91.162(2)°, V = 2676.73(9) Å^3, Z = 4, T = 100.02(10) K, μ(Cu Kα) = 0.973 mm^{-1}, $Dcalc$ = 1.143 g/cm^3, 15574 reflections measured (11.144° ≤ 2Θ ≤ 136.488°), 4879 unique (R_{int} = 0.0565, R_{sigma} = 0.0555) which were used in all calculations. The final R_1 was 0.0340 (I > 2σ(I)) and wR_2 was 0.0818 (all data).

Refinement model description

Number of restraints - 1, number of constraints - unknown.

Details:

1. Fixed Uiso
 At 1.2 times of:
  All C(H) groups, All C(H,H) groups
 At 1.5 times of:
  All C(H,H,H) groups, All O(H) groups
2.a Ternary CH refined with riding coordinates:
 C00B(H00D)

2.b Secondary CH2 refined with riding coordinates:
C00D(H00E,H00F)
2.c Aromatic/amide H refined with riding coordinates:
C008(H008), C009(H009), C00J(H00J), C00K(H00K), C00M(H00M), C00O(H00P), C00P(H00Q), C00Q(H00R), C00T(H00Y), C00V(H00), C00W(H1)
2.d Idealised Me refined as rotating group:
C005(H00A,H00B,H00C), C00I(H00G,H00H,H00I), C00N(H00L,H00N,H00O), C00R(H00S,
H00T,H00U), C00S(H00V,H00W,H00X), C00U(H00Z,H,HA), C00X(H2,HB,HC)
2.e Idealised tetrahedral OH refined as rotating group:
O003(H003)

Table S4 Crystal data and structure refinement for [**d_9-*(R,R)*-16**]

Identification code	[**d_9-*(R,R)*-16**]
Empirical formula	$C_{29}H_{36}O_3Si$
Formula weight	460.67
Temperature/K	100.02(10)
Crystal system	monoclinic
Space group	C2
a/Å	15.0513(3)
b/Å	13.3427(2)
c/Å	13.3314(3)
α/°	90
β/°	91.162(2)
γ/°	90
Volume/Å^3	2676.73(9)
Z	4
ρ_{calc}g/cm^3	1.143
μ/mm^{-1}	0.973
F(000)	992.0
Crystal size/mm^3	? × ? × ?
Radiation	Cu Kα (λ = 1.54184)
2Θ range for data collection/°	11.144 to 136.488

Index ranges	-18 ≤ h ≤ 17, -16 ≤ k ≤ 16, -16 ≤ l ≤ 15
Reflections collected	15574
Independent reflections	4879 [R_{int} = 0.0565, R_{sigma} = 0.0555]
Data/restraints/parameters	4879/1/306
Goodness-of-fit on F^2	1.040
Final R indexes [I>=2σ (I)]	R_1 = 0.0340, wR_2 = 0.0803
Final R indexes [all data]	R_1 = 0.0369, wR_2 = 0.0818
Largest diff. peak/hole / e Å^{-3}	0.30/-0.25
Flack parameter	0.033(17)

Table S5 Fractional Atomic Coordinates (×10^4) and Equivalent Isotropic Displacement Parameters (Å^2×10^3) for [d$_9$-*(R,R)*-16]. U_{eq} is defined as 1/3 of of the trace of the orthogonalised U_{IJ} tensor.

Atom	*x*	*y*	*z*	U(eq)
Si01	2298.1(4)	6110.4(5)	1523.3(5)	17.47(16)
O002	1883.2(11)	6902.5(13)	2352.7(13)	19.5(4)
O003	2821.1(13)	5784.6(14)	6147.2(14)	25.3(4)
O004	1337.3(13)	2292.3(14)	4867.4(15)	27.8(4)
C005	711.2(17)	7616.1(19)	3313(2)	21.8(5)
C006	2270.6(18)	6808(2)	283(2)	23.4(6)
C007	1626.2(18)	4929.1(19)	1572.0(19)	21.7(5)
C008	2540.0(17)	3419.6(18)	4831.5(19)	20.0(5)
C009	3895.0(17)	6293.9(19)	2708(2)	22.1(5)
C00A	2870.2(16)	4325.3(18)	5045.2(18)	17.6(5)
C00B	1209.4(16)	6659.9(18)	3056.9(19)	17.4(5)
C00C	1561.7(17)	4688(2)	6175(2)	22.2(5)
C00D	1060.1(17)	3911(2)	5539(2)	23.4(6)
C00E	3477.0(18)	5814.7(19)	1898(2)	22.8(5)
C00F	1904.2(16)	5721.8(18)	4659(2)	19.1(5)

C00G	1600.0(15)	6159(2)	3944.4(18)	18.4(5)
C00H	2296.2(16)	5152.0(19)	5512.9(19)	19.2(5)
C00I	1980.3(19)	4197(2)	7108(2)	26.0(6)
C00J	1912(2)	4124(2)	2171(2)	29.3(6)
C00K	4776.3(17)	6086(2)	2976(2)	28.5(6)
C00L	1634.1(17)	3129(2)	5068(2)	20.4(5)
C00M	783.5(19)	4847(2)	1118(2)	28.6(6)
C00N	3800.8(17)	4618(2)	4810(2)	25.7(6)
C00O	3971(2)	5111(2)	1364(2)	34.4(7)
C00P	256(2)	4005(2)	1246(2)	36.0(7)
C00Q	1382(2)	3278(2)	2295(2)	37.6(8)
C00R	911(2)	5496(2)	6509(2)	31.8(6)
C00S	2913(2)	7697(2)	396(2)	35.3(7)
C00T	5256.1(19)	5407(2)	2428(2)	35.1(7)
C00U	2563(3)	6144(3)	-576(2)	43.8(8)
C00V	556(2)	3224(2)	1824(2)	38.8(8)
C00W	4856(2)	4915(3)	1626(3)	40.8(8)
C00X	1344(2)	7233(3)	38(3)	46.7(9)

Table S6 Anisotropic Displacement Parameters (Å^2×10^3) for [d$_9$-*(R,R)*-16]. The Anisotropic displacement factor exponent takes the form: $-2\pi^2[h^2a^{*2}U_{11}+2hka^*b^*U_{12}+\ldots]$.

Atom	U_{11}	U_{22}	U_{33}	U_{23}	U_{13}	U_{12}
Si01	18.8(3)	16.1(3)	17.5(3)	1.0(3)	-0.5(2)	-2.3(3)
O002	20.8(9)	16.6(8)	21.3(9)	0.9(7)	3.6(7)	-4.1(7)
O003	30.8(10)	22.0(9)	22.9(10)	-0.1(7)	-6.0(8)	-0.7(7)
O004	25.9(10)	25.6(10)	31.7(11)	1.4(8)	-6.8(8)	-3.8(8)
C005	19.7(12)	19.3(12)	26.2(14)	2.0(10)	-2.7(10)	2.5(10)
C006	27.5(14)	20.9(12)	21.8(14)	2.7(10)	-1.2(10)	-6.0(10)
C007	29.4(14)	18.4(12)	17.4(12)	-2.3(10)	5.2(10)	-3.5(10)

C008	18.2(12)	22.0(13)	19.8(13)	1.1(10)	-0.5(10)	6.2(10)
C009	22.6(13)	20.6(13)	23.1(12)	5.5(10)	1.6(10)	-4.6(10)
C00A	15.4(11)	22.5(12)	15.0(12)	4.5(9)	1.2(9)	4.7(10)
C00B	15.8(11)	17.3(12)	19.1(12)	0.8(9)	1.6(9)	-2.6(9)
C00C	21.6(12)	22.5(12)	22.7(13)	4.0(10)	4.0(10)	7.5(10)
C00D	15.7(12)	25.4(13)	29.1(15)	9.1(11)	2.2(10)	4.3(10)
C00E	23.8(13)	21.6(12)	23.1(13)	3.1(10)	1.0(10)	-1.5(10)
C00F	19.0(12)	17.1(11)	21.4(13)	0.2(10)	1.4(10)	1.8(9)
C00G	16.2(10)	16.6(10)	22.3(12)	-1.2(11)	1.0(9)	0.2(10)
C00H	20.1(12)	18.7(12)	18.8(12)	0.7(10)	-0.7(9)	3.8(9)
C00I	30.1(14)	26.1(13)	21.9(13)	6.2(11)	6.4(11)	8.8(11)
C00J	44.1(17)	22.8(13)	20.9(14)	0.5(11)	3.2(12)	-2.7(12)
C00K	24.3(12)	33.0(14)	28.1(13)	10.9(13)	-4.1(10)	-8.3(13)
C00L	18.2(12)	22.6(12)	20.2(13)	4.4(10)	-4.4(10)	0.7(10)
C00M	29.1(15)	28.3(14)	28.7(15)	-2.0(11)	3.0(11)	-9.5(11)
C00N	18.9(12)	29.2(14)	29.2(15)	7.6(11)	3.9(11)	3.1(11)
C00O	34.7(16)	35.5(15)	32.7(16)	-5.1(13)	-4.2(12)	9.3(13)
C00P	38.2(17)	35.8(16)	34.3(17)	-5.4(13)	8.4(13)	-17.5(13)
C00Q	64(2)	23.6(14)	25.8(15)	4.9(12)	11.6(14)	-4.2(14)
C00R	34.4(15)	31.7(15)	29.6(15)	4.9(12)	9.0(12)	17.5(12)
C00S	49.4(19)	32.5(15)	23.8(15)	7.1(13)	-0.4(13)	-20.5(14)
C00T	22.7(14)	42.4(17)	40.3(18)	17.6(14)	-0.3(13)	4.6(12)
C00U	77(2)	37.1(16)	17.4(13)	-1.5(14)	5.2(14)	-9.3(19)
C00V	55(2)	31.4(15)	30.8(16)	-7.0(13)	15.6(14)	-23.3(14)
C00W	35.8(17)	45.1(18)	41.7(19)	1.3(15)	3.9(14)	18.0(14)
C00X	32.3(17)	58(2)	50(2)	30.3(18)	-5.4(14)	1.8(15)

Table S7 Bond Lengths for [d$_9$-*(R*,*R*)-16].

Atom	Atom	Length/Å		Atom	Atom	Length/Å
Si01	O002	1.6605(18)		C00A	C00N	1.493(4)
Si01	C006	1.897(3)		C00B	C00G	1.471(4)
Si01	C007	1.874(3)		C00C	C00D	1.528(4)
Si01	C00E	1.876(3)		C00C	C00H	1.557(4)
O002	C00B	1.433(3)		C00C	C00I	1.530(4)
O003	C00H	1.423(3)		C00C	C00R	1.530(4)
O004	C00L	1.230(3)		C00D	C00L	1.501(4)
C005	C00B	1.522(3)		C00E	C00O	1.401(4)
C006	C00S	1.536(4)		C00F	C00G	1.200(4)
C006	C00U	1.520(4)		C00F	C00H	1.482(4)
C006	C00X	1.534(4)		C00J	C00Q	1.394(4)
C007	C00J	1.401(4)		C00K	C00T	1.377(5)
C007	C00M	1.398(4)		C00M	C00P	1.388(4)
C008	C00A	1.335(4)		C00O	C00W	1.395(5)
C008	C00L	1.458(4)		C00P	C00V	1.367(5)
C009	C00E	1.394(4)		C00Q	C00V	1.383(5)
C009	C00K	1.395(4)		C00T	C00W	1.383(5)
C00A	C00H	1.541(3)				

Table S8 Bond Angles for [d$_9$-*(R*,*R*)-16].

Atom	Atom	Atom	Angle/°		Atom	Atom	Atom	Angle/°
O002	Si01	C006	105.44(11)		C00I	C00C	C00H	110.2(2)
O002	Si01	C007	107.56(10)		C00R	C00C	C00H	110.5(2)
O002	Si01	C00E	108.85(11)		C00R	C00C	C00I	108.7(2)
C007	Si01	C006	116.13(12)		C00L	C00D	C00C	114.9(2)
C007	Si01	C00E	108.79(12)		C009	C00E	Si01	121.3(2)
C00E	Si01	C006	109.81(12)		C009	C00E	C00O	117.7(3)

C00B	O002	Si01	124.97(15)		C00O	C00E	Si01	121.0(2)
C00S	C006	Si01	106.92(18)		C00G	C00F	C00H	177.7(3)
C00U	C006	Si01	111.67(19)		C00F	C00G	C00B	177.8(3)
C00U	C006	C00S	109.5(3)		O003	C00H	C00A	110.9(2)
C00U	C006	C00X	109.3(3)		O003	C00H	C00C	106.9(2)
C00X	C006	Si01	111.8(2)		O003	C00H	C00F	111.1(2)
C00X	C006	C00S	107.6(3)		C00A	C00H	C00C	110.8(2)
C00J	C007	Si01	120.3(2)		C00F	C00H	C00A	105.9(2)
C00M	C007	Si01	122.4(2)		C00F	C00H	C00C	111.3(2)
C00M	C007	C00J	116.9(3)		C00Q	C00J	C007	121.2(3)
C00A	C008	C00L	122.7(2)		C00T	C00K	C009	120.1(3)
C00E	C009	C00K	121.3(3)		O004	C00L	C008	122.1(2)
C008	C00A	C00H	121.7(2)		O004	C00L	C00D	120.8(2)
C008	C00A	C00N	122.6(2)		C008	C00L	C00D	117.0(2)
C00N	C00A	C00H	115.7(2)		C00P	C00M	C007	121.8(3)
O002	C00B	C005	108.41(19)		C00W	C00O	C00E	120.9(3)
O002	C00B	C00G	110.67(19)		C00V	C00P	C00M	120.1(3)
C00G	C00B	C005	113.0(2)		C00V	C00Q	C00J	120.0(3)
C00D	C00C	C00H	107.7(2)		C00K	C00T	C00W	119.9(3)
C00D	C00C	C00I	110.6(2)		C00P	C00V	C00Q	120.0(3)
C00D	C00C	C00R	109.1(2)		C00T	C00W	C00O	120.2(3)

Table S9 Torsion Angles for [d9-*(R*,*R*)-16].

A	B	C	D	Angle/°		A	B	C	D	Angle/°
Si01	O002	C00B	C005	-153.40(17		C00C	C00D	C00L	C008	-29.1(3)
Si01	O002	C00B	C00G	82.1(2)		C00D	C00C	C00H	O003	-173.35(19)
Si01	C007	C00J	C00Q	173.7(2)		C00D	C00C	C00H	C00A	-52.4(3)
Si01	C007	C00M	C00P	-173.3(2)		C00D	C00C	C00H	C00F	65.1(3)
Si01	C00E	C00O	C00W	178.1(3)		C00E	Si01	O002	C00B	-110.47(19)
O002	Si01	C006	C00S	65.0(2)		C00E	Si01	C006	C00S	-52.1(2)
O002	Si01	C006	C00U	-175.3(2)		C00E	Si01	C006	C00U	67.6(2)
O002	Si01	C006	C00X	-52.5(2)		C00E	Si01	C006	C00X	-169.6(2)
O002	Si01	C007	C00J	-95.0(2)		C00E	Si01	C007	C00J	22.8(3)
O002	Si01	C007	C00M	77.5(2)		C00E	Si01	C007	C00M	-164.7(2)
O002	Si01	C00E	C009	-4.9(2)		C00E	C009	C00K	C00T	1.1(4)
O002	Si01	C00E	C00O	175.8(2)		C00E	C00O	C00W	C00T	0.7(5)
C006	Si01	O002	C00B	131.76(19)		C00H	C00C	C00D	C00L	53.8(3)
C006	Si01	C007	C00J	147.2(2)		C00I	C00C	C00D	C00L	-66.7(3)
C006	Si01	C007	C00M	-40.3(3)		C00I	C00C	C00H	O003	-52.7(3)
C006	Si01	C00E	C009	110.0(2)		C00I	C00C	C00H	C00A	68.3(3)
C006	Si01	C00E	C00O	-69.2(3)		C00I	C00C	C00H	C00F	-174.2(2)
C007	Si01	O002	C00B	7.2(2)		C00J	C007	C00M	C00P	-0.5(4)
C007	Si01	C006	C00S	-176.0(2)		C00J	C00Q	C00V	C00P	-0.7(5)
C007	Si01	C006	C00U	-56.3(2)		C00K	C009	C00E	Si01	-179.0(2)
C007	Si01	C006	C00X	66.4(3)		C00K	C009	C00E	C00O	0.3(4)
C007	Si01	C00E	C009	-121.9(2)		C00K	C00T	C00W	C00O	0.7(5)
C007	Si01	C00E	C00O	58.9(3)		C00L	C008	C00A	C00H	-3.2(4)
C007	C00J	C00Q	C00V	-0.2(5)		C00L	C008	C00A	C00N	179.5(2)
C007	C00M	C00P	C00V	-0.3(5)		C00M	C007	C00J	C00Q	0.8(4)
C008	C00A	C00H	O003	148.3(2)		C00M	C00P	C00V	C00Q	0.9(5)

C008	C00A	C00H	C00C	29.7(3)		C00N	C00A	C00H	O003	-34.2(3)
C008	C00A	C00H	C00F	-91.1(3)		C00N	C00A	C00H	C00C	-152.7(2)
C009	C00E	C00O	C00W	-1.2(4)		C00N	C00A	C00H	C00F	86.4(3)
C009	C00K	C00T	C00W	-1.6(4)		C00R	C00C	C00D	C00L	173.8(2)
C00A	C008	C00L	O004	180.0(2)		C00R	C00C	C00H	O003	67.5(3)
C00A	C008	C00L	C00D	2.1(4)		C00R	C00C	C00H	C00A	-171.6(2)
C00C	C00D	C00L	O004	152.9(2)		C00R	C00C	C00H	C00F	-54.0(3)

Table S10 Hydrogen Atom Coordinates (Å×10^4) and Isotropic Displacement Parameters (Å^2×10^3) for [d$_9$-*(R*,*R*)-16].

Atom	*x*	*y*	*z*	U(eq)
H003	3069.37	6208.21	5806.36	38
H00A	1122.72	8104.45	3574.72	33
H00B	273.52	7471.71	3806.12	33
H00C	423.16	7875.17	2718.12	33
H008	2902.7	2955.07	4518.99	24
H009	3580.1	6761.62	3076.29	27
H00D	789.16	6195.57	2732.04	21
H00E	631.63	3577.69	5958.99	28
H00F	730.86	4257.82	5011.61	28
H00G	1521.35	3917.75	7512.36	39
H00H	2304.92	4690.92	7489.29	39
H00I	2377.35	3673.94	6907.75	39
H00J	2466.02	4155.11	2492.57	35
H00K	5040.03	6405.56	3526.07	34
H00M	570.86	5369.95	720.13	34
H00L	4138.21	4700.77	5422.84	39
H00N	3795.3	5236.65	4442.71	39
H00O	4067.21	4103.04	4412.01	39

H00P	3706.14	4770.84	827.72	41
H00Q	-303.39	3971.84	938.06	43
H00R	1584.17	2749.86	2693.52	45
H00S	626.53	5791.84	5929.96	48
H00T	1228.42	6004.31	6879.69	48
H00U	470.42	5200.94	6927.38	48
H00V	2740.2	8105.19	951.97	53
H00W	2895.2	8090.77	-207.23	53
H00X	3505.8	7451.83	513.03	53
H00Y	5847.99	5280.46	2597.59	42
H00Z	3157.57	5912.37	-445.51	66
H	2544.48	6520.39	-1189.99	66
HA	2169.78	5579.02	-636.24	66
H00	205.16	2657.18	1900.81	47
H1	5178.18	4451	1260.01	49
H2	933.41	6691.9	-69.87	70
HB	1368.86	7636.43	-557.45	70
HC	1151.28	7638.01	587.56	70
